# The Vacuolar Protein Sorting-38 Subunit of the *Arabidopsis* Phosphatidylinositol-3-Kinase Complex Plays Critical Roles in Autophagy, Endosome Sorting, and Gravitropism

**DOI:** 10.3389/fpls.2018.00781

**Published:** 2018-06-18

**Authors:** Fen Liu, Weiming Hu, Richard D. Vierstra

**Affiliations:** ^1^Department of Biology, Washington University in St. Louis, St. Louis, MO, United States; ^2^South China Botanical Garden, Chinese Academy of Sciences, Guangzhou, China

**Keywords:** *Arabidopsis*, phosphatidylinositol 3-phosphate (PtdIn-3P), vesicle trafficking, pollen, autophagy, gravitropism, polar auxin transport

## Abstract

The family of phosphatidylinositols (PtdIns) plays essential roles in membrane identity and intracellular trafficking events. In animals and yeast, PtdIn-3-phosphate, which is particularly important for endosomal sorting, lysosomal/vacuolar transport and autophagy, is assembled by two conserved kinase complexes comprised of the catalytic VACUOLAR PROTEIN SORTING (VPS)-34 subunit, along with VPS15, AUTOPHAGY-RELATED (ATG)-6, and either ATG14 (complex I) or VPS38 (complex II). Here, we describe the *Arabidopsis* ortholog of VPS38 and show by interaction assays that it assembles into a tetrameric PtdIn-3 kinase complex II. Plants missing VPS38 are viable but have dampened pollen germination and heightened seed abortion, and display a dwarf rosette phenotype, with defects in leaf and vascular development and sucrose sensing. *vps38* seeds accumulate irregular protein storage vesicles and suppress processing of storage proteins into their mature forms. Consistent with a role for PtdIn-3-phosphate in autophagy, *vps38* mutants are hypersensitive to nitrogen and fixed-carbon starvation and show reduced autophagic transport of cargo into vacuoles. *vps38* seedlings also have dampened root gravitropism, which is underpinned by aberrant vectoral auxin transport likely caused by defects in plasma membrane/endosome cycling of the PIN-FORMED family of auxin transporters necessary for asymmetric cell elongation. Collectively, this study places VPS38 and its class-III PtdIn-3 kinase complex at the nexus of numerous endosomal trafficking events important to plant growth and development.

## Introduction

Cellular trafficking and membrane/organelle identity are determined by the combined actions of specific lipids and proteins on each membrane surface. Key effectors in these processes are the phosphatidylinositol (PtdIn) family of lipids, which through phosphorylation at one or more positions of their *myo*-inositol head group generates an array of PtdIns offering unique membrane signatures (Balla, 2013; Noack and Jaillais, 2017). Consequently, through the combined action of kinases and phosphatases, a dynamic set of mono-, bis-, and tris-phosphate-containing PtdIns modified at the 3′, 4′, and/or 5′ positions are assembled that provide spatial and temporal control of protein trafficking and endosomal sorting. Ultimately, these species are recognized by specific PtdIn-binding proteins that promote vesicle enclosure, transport and fusion, and eventually help distinguish specific cytoplasmic compartments. In plants, a central trafficking route involves release of cargo-containing vesicles from the endoplasmic reticulum or plasma membrane and entry of these vesicles into the *trans*-Golgi network (TGN) that includes early endosomes (Gerth et al., 2017; Noack and Jaillais, 2017). Subsequently, the vesicles enter multivesicular bodies (MVBs) that sort and release them as late endosomes for transport of the cargo to appropriate compartments where they are deposited by vesicle fusion.

**FIGURE 1:**
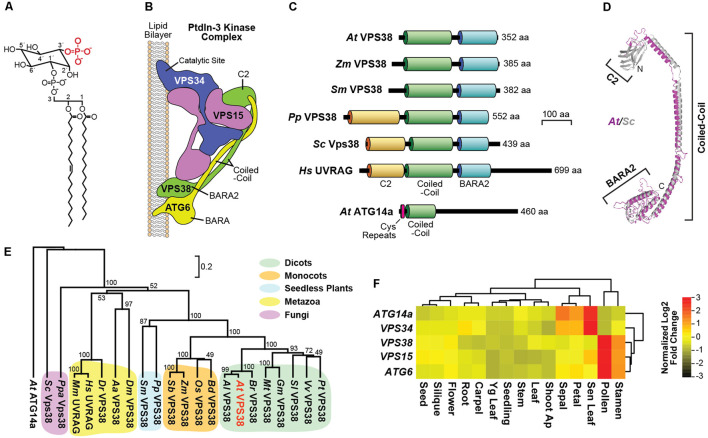
Description of the *Arabidopsis* class-III PtdIn-3 kinase complex and architecture and expression of the VPS38 subunit. **(A)** Chemical structure of a representative PtdIn-3P. The 3′ phosphate group is highlighted in red. **(B)** Subunit organization of the yeast class-III PtdIns-3 kinase complex II based on a crystallographic structure [PDB entry 5DFZ (Rostislavleva et al., 2015)]. The VPS34, VPS15, ATG6 (VPS30/Beclin1), and VPS38 subunits are labeled along with relevant domains. **(C)** Architecture and domain organization of members in the VPS38 family. The lipid-binding C2, coiled-coil, and BARA2 domains are highlighted. The amino acid (aa) length of each protein is indicated. *Arabidopsis* ATG14a, with its pair of cysteine repeats, is included for reference. **(D)** The predicted 3D model for *Arabidopsis* VPS38 (magenta) based on the yeast Vps38 structure (gray). The *Arabidopsis* sequence was threaded into the yeast structure (Rostislavleva et al., 2015) using SWISS-MODEL, and the ribbon diagrams were superimposed. N, amino-terminus; C, carboxy-terminus. **(E)** Phylogenetic tree of the VPS38/UVRAG family. The phylogenetic relationships based on protein sequences were assessed by MEGA 7, using the unweighted pair group method with arithmetic mean, and visualized with TreeView. *Arabidopsis* VPS38 is highlighted in red. The scale bar indicates the percent distance. ClustalX alignment of the VPS38 sequences based on identity/similarity is shown in Supplementary Figure S1. Species abbreviations are: *Aa*, *Aedes aegypti*; *Al*, *Arabidopsis lyrata*; *At*, *Arabidopsis thaliana; Bd*, *Brachypodium distachyon*; *Br*, *Brassica rapa*; *Dm*, *Drosophila melanogaster*; *Dr*, *Danio rerio*; *Gm*, *Glycine max*; *Hs*, *Homo sapiens*; *Mt*, *Medicago truncatula*; *Os*, *Oryza sativa*; *Pp*, *Physcomitrella patens*; *Ppa: Pichia pastoris*; *Pt*, *Populus trichocarpa*; *Sb*, *Sorghum bicolor*; *Sc*, *Saccharomyces cerevisiae; Sl*, *Solanum lycopersicum*; *Sm*, *Selaginella moellendorffii*; *Vv*, *Vitis vinifera*; and *Zm*, *Zea mays*. **(F)** Expression heat map of transcripts encoding subunits of the *Arabidopsis* class-III PtdIn-3 kinase complex. The mRNA abundances were obtained from GENEVESTIGATOR (https://www.genevestigator.com) and clustered by tissue distributions and expression patterns. The relative expression of each gene presented in *Z*-score was normalized against the mean value by Log2 transformation. Values for the *ATG14a* mRNA were included as a reference.

Although representing a minor fraction of the lipid pool, PtdIn bearing a phosphate moiety at the 3′ position (PtdIn-3P; **Figure 1A**) has emerged as a central player in endocytosis, endosomal sorting, and autophagy (Di Sansebastiano et al., 2017; Gerth et al., 2017; Noack and Jaillais, 2017). Here, PtdIn-3P is detected by a collection of proteins containing Fab1/YOTB/Vac1/EEA1 (FYVE) zinc finger or Phox-HOMOLOGY domains that recognize the inositol-3P moiety. For example, the *Arabidopsis thaliana* FYVE DOMAIN PROTEIN REQUIRED FOR ENDOSOMAL SORTING (FREE)-1 uses the FYVE domain to bind emerging autophagy membranes, and, together with another PtdIn-3P-binding protein SH3-DOMAIN CONTAINING PROTEIN (SH3P)-2, helps direct membrane curvature and autophagic vesicle delivery (Zhuang et al., 2013; Gao et al., 2014, 2015).

In animals, PtdIn-3P is synthesized by three distinct kinase complexes with unique subunit compositions (Balla, 2013), but only the class III complex is evident in plants (Gerth et al., 2017; Noack and Jaillais, 2017). This class III kinase is assembled with VACUOLAR PROTEIN SORTING (VPS)-34, which provides the phosphotransferase activity, and two core accessory subunits VPS15 and ATG6 (VPS30 in yeast or Beclin1 in mammals). Its functions are further discriminated by a fourth subunit, either ATG14 for the complex-I isoform, or VPS38 (UV RADIATION RESISTANCE ASSOCIATED GENE (UVRAG) in mammals) for the complex-II isoform. Whereas, VPS38/UVRAG appears to direct trafficking events required for vacuolar protein sorting, ATG14 appears specific for autophagic vesicle development (Kihara et al., 2001; Itakura et al., 2008). Recent structural analysis of the entire yeast class-III complex (Rostislavleva et al., 2015) revealed that it assumes a V shape with one arm positioning the catalytic Vps34/Vps15 heterodimer to modify membrane-embedded PtdIn at the 3′ position, and a second arm created by Atg6 (Vps30/Beclin1) and Vps38 that intertwines through an extended parallel coiled-coil region and binds to the membrane at a second site via their respective Beta-Alpha-Repeated, Autophagy-Specific (BARA) and BARA2 domains (**Figure 1B**).

Numerous studies on predicted core subunits of the *Arabidopsis* class-III PtdIn-3 kinase concluded that a similar complex is assembled in plants and showed that this kinase, and by inference that its PtdIn-3P product, are essential. Notably, homozygous mutants missing VPS34 (Lee et al., 2008a,b), VPS15 (Xu et al., 2011; Wang et al., 2012), and ATG6 (VPS30/Beclin1) (Fujiki et al., 2007; Qin et al., 2007; Harrison-Lowe and Olsen, 2008) cannot be generated due to defects in male gamete development. While the mutant pollen appears viable, a strong block in pollen germination prevents transmission of the mutant allele and creation of homozygous progeny even though the female gametophyte appears normal. Aberrant seedling development was also reported for heterozygous *atg6* plants (Harrison-Lowe and Olsen, 2008) or homozygous *atg6* plants rescued with low levels of an ATG6-GFP fusion (Qin et al., 2007), indicating that other aspects of *Arabidopsis* development require PtdIn-3P and this kinase activity as well, but can only be observed with weak alleles. Roles in autophagy, cellulose synthesis, gravitropism, and the vacuolar sorting of seed storage proteins have also been proposed based on the effects of the class-III PtdIn-3 kinase-selective inhibitors – wortmannin and LY294002 on plant development (Takatsuka et al., 2004; Joo et al., 2005; Takáč et al., 2013; Fujimoto et al., 2015).

Given that the yeast and mammalian Vps38/UVRAG and ATG14 subunits confer non-redundant functions to the class-III PtdIn-3 kinase (Kihara et al., 2001; Itakura et al., 2008), we considered it feasible that the corresponding homozygous mutants would be individually viable in *Arabidopsis*, thus enabling us to study the functions of this kinase complex and its PtdIn-3P product throughout plant development. Here, we describe *Arabidopsis* mutants eliminating VPS38 specific for complex II. Unlike mutants affecting genes encoding the three core subunits of the class-III PtdIn-3 kinase complex (VPS34, VPS15, and ATG6), homozygous *vps38* mutants survive, and even though pollen germination is dampened, reproduction is possible.

Studies of homozygous mutant plants connected VPS38, and presumably the entire kinase complex, to leaf and seed development, and survival under nitrogen- and fixed-carbon-limiting growth conditions. Whereas Atg14 has been specifically connected to autophagy in yeast (Kihara et al., 2001; Diao et al., 2015), we found that autophagy is also compromised in *Arabidopsis* mutants missing VPS38, indicating that the plant complex-II isoform is also engaged in this recycling process. Surprisingly, *Arabidopsis vps38* mutants have suppressed gravitropism, which are underpinned by defects in auxin transport. Problems in the plasma membrane to endosome cycling of the PIN-FORMED (PIN) auxin efflux carriers necessary for tropic curvature were observed, thus connecting VPS38 to the endocytosis and vesicular trafficking pathways that control these carriers (Kleine-Vehn et al., 2008; Di Sansebastiano et al., 2014; Adamowski and Friml, 2015). Together with a companion paper by Lee et al. (2018), genetic analysis of the non-essential VPS38 subunit reveals that the class-III PtdIn-3 kinase complex has pleiotropic roles in membrane identity and intracellular trafficking in plants.

## Results

### Identification of *Arabidopsis* VPS38

Given the potential importance of PtdIn-3P to intracellular trafficking in plants and the essential nature of the *Arabidopsis* VPS34, VPS15, and ATG6 (VPS30/Beclin1) subunits that generate this PtdIn (see **Figure 1B**), we sought to investigate its functions through the analysis of *Arabidopsis* counterparts of the human and yeast ATG14 and Vps38/UVRAG subunits that specifically define the class-III PtdIn-3 kinase complexes I and II, respectively (Kihara et al., 2001; Itakura et al., 2008). BLASTP searches of the *A. thaliana* Columbia (Col)-0 genome database using the non-plant sequences as queries identified possible orthologs of each. Two closely related *Arabidopsis* loci encode 460- and 477-residue relatives of ATG14, which share 21–34% amino acid sequence identity to their non-plant brethren and contain the signature N-terminal cysteine-repeats followed by a coiled-coil domain (Diao et al., 2015). Both *Arabidopsis* proteins were previously described in GenBank as a possible DNA-directed RNA polymerase II, but were reannotated here as ATG14a (At1g77890) and ATG14b (At4g08540) based on our homology data (**Figure 1C**) and demonstrated interactions between ATG14a/b and ATG6 (data not shown).

One *Arabidopsis* locus was discovered to encode a 352-residue relative of VPS38 (At2g32760) with 16 and 24% sequence identity to yeast Vps38 and human UVRAG, respectively. This sequence included the signature coiled-coil and BARA2 domains found in their non-plant counterparts (Rostislavleva et al., 2015), but was notably absent the N-terminal lipid-binding C2 domain (**Figure 1C**). The predicted 3D structure of *Arabidopsis* VPS38 superposed well with the empirically determined structure for the region downstream of the C2 domain in yeast Vps38 (Rostislavleva et al., 2015), including close overlap of the coiled-coil and BARA2 folds (**Figure 1D**).

Subsequent searches with *Arabidopsis* VPS38 identified a large collection of likely orthologs in other plant species (35–100% amino acid sequence identity), ranging from seedless plants to both monocots and dicots (Supplementary Figure S1). These sequences clustered phylogenetically into several well-supported clades distinct from the fungal and animal versions and separate from ATG14a/b (**Figure 1E**). Surprisingly, whereas the predicted domain architectures of seed plant VPS38 sequences were missing the C2 domain that is prevalent outside of plants, the *Physcomitrella patens* but not the *Selaginella moellendorffii* VPS38 sequence included this domain (**Figure 1C**), suggesting that the plant versions lost the C2 region sometime during the evolutionary transition from bryophytes to ferns.

### Expression Patterns of *VPS38*

Expression patterns gathered from the extensive *A. thaliana* Col-0 microarray datasets available in GENEVESTIGATOR^1^ and the *Arabidopsis* eFP Browser 2.0^2^ showed that the *VPS38* and *ATG14a* loci are ubiquitously expressed at low levels in most tissues throughout *Arabidopsis* development (**Figure 1F** and Supplementary Figure S2). (No expression data were available for *ATG14b*). Strong expression was apparent in pollen and stamens for *VPS38*, which is consistent with a prominent role for the class-III PtdIn-3 kinase complex II in pollen development and germination (Fujiki et al., 2007; Qin et al., 2007; Harrison-Lowe and Olsen, 2008; Lee et al., 2008b; Xu et al., 2011; Wang et al., 2012). Conversely, *ATG14a* was best expressed in senescing leaves and ephemeral petals and sepals, consistent with the roles for ATG14 and the rest of this PtdIn-3 kinase complex I during cell death and autophagy in other organisms (Cao and Klionsky, 2007; Lindmo et al., 2008; Jaber et al., 2012; Diao et al., 2015). Interestingly, the transcript patterns of *VPS38* best correlated with those encoding the accessory VPS15 and ATG6 subunits shared by both the I and II complexes, while the transcript patterns of *ATG14a* best correlated with those encoding the catalytic VPS34 subunit (**Figure 1F** and Supplementary Figure S2). Collectively, the expression data suggest that VPS38 and ATG14 have distinct roles in *Arabidopsis* growth and development.

### VPS38 Interacts With Other Members of Class-III PtdIn-3 Kinase Complex

**FIGURE 2:**
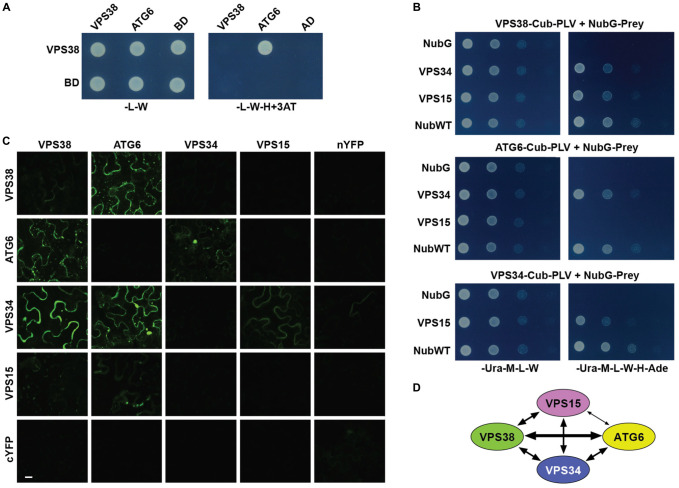
*Arabidopsis* VPS38 interacts with other subunits of class-III PtdIns-3 kinase complex. **(A)** Pairwise Y2H assays using the GAL4-based system showing that VPS38 interacts with ATG6. VSPS38 fused to the N terminus of the DNA-binding domain (BD) and ATG6 fused to the N terminus of the activation domain (AD) were co-expressed in yeast and tested for binding by growth on synthetic complete medium lacking leucine, tryptophan, and histidine (-L-W-H), and containing 3-amino-1,2,4-triazole (+3AT). Viability of the cells was confirmed by growth on medium lacking leucine and tryptophan (-L-W). **(B)** Pairwise Y2H assays by the split-ubiquitin mating system showing interactions among VPS38, ATG6, VPS15, and VPS34. Each full-length protein was expressed as a fusion to either Cub-PLV as bait or NubG as prey, and co-expressed in diploid yeast cells. Positive interactions were determined by growth of twofold serial dilutions on synthetic complete medium lacking uracil, methionine, leucine, tryptophan, histidine, and adenine (-Ura-M-L-W-H-Ade). The empty NubG and NubWT vectors were used as negative and positive controls, respectively. Viability of the cells was confirmed by growth on synthetic complete medium lacking uracil, methionine, leucine, and tryptophan (-Ura-M-L-W). **(C)** Pairwise BiFC assays showing the interactions among VPS38, ATG6, VPS15, and VPS34 *in planta*. Each full-length protein was expressed as a fusion to either N-terminal fragment (nYFP) or C-terminal fragment (cYFP) of YFP and then transiently co-expressed in *N. benthamiana* leaf epidermal cells. Appearance of the fluorescent signals was observed by confocal microscopic analysis 36 h after infiltration. Scale bar = 20 μm. **(D)** Schematic of the interactions detected among VPS38, ATG6, VPS15, and VPS34. The arrow thickness is an estimate of binding strength based on all interaction assays. The solid and dashed lines indicate interactions that were demonstrated by both Y2H and BiFC, or by just one of the methods, respectively.

Given the low sequence homology of *Arabidopsis* VPS38 with its human and yeast counterparts, we attempted to confirm that it indeed assembles into a class-III PtdIn-3 kinase complex II by interaction assays. As a first attempt, we tested by standard Gal4-based, yeast two-hybrid (Y2H) assays whether VPS38 interacts with ATG6, based on the yeast model that both subunits intertwine through their coiled-coil regions (see **Figure 1B**). As shown in **Figure 2A**, binding between VPS38 and ATG6 was detected with ATG6 in the Gal4 activation-domain orientation. (ATG6 in the Gal4 DNA-binding-domain orientation self-activated in the assay). To provide greater clarity, we adopted a split ubiquitin interaction system to test pairwise binding of all the predicted complex-II subunits (VPS34, VPS15, ATG6, and VPS38); this assay was designed for membrane-associated proteins based on the ubiquitin-mediated proteolytic release of the membrane-anchored, Protein A/LexA/VP16 transcriptional activator (Stagljar et al., 1998). Interactions between VPS38 and either VPS34 or VPS15, between ATG6 and VPS34, and between VPS34 and VPS15 were obvious from growths on selection medium (**Figure 2B**).

And finally, we exploited Bimolecular Fluorescence Complementation (BiFC) assays that express split-eYFP fusions in *Nicotiana benthamiana* leaves to assess interactions *in planta*. Here, binding of VPS38 to ATG6, binding of VPS34 to either VPS38, VPS15 or ATG6, and binding of VPS15 to ATG6 were detected by diffuse cytoplasmic fluorescence 36 h after co-infiltration of the eYFP plasmids (**Figure 2C**). In total, the interaction studies confirmed the identity of VPS38 despite low sequence homology to its non-plant relatives, and were consistent with the 3D structure of the yeast class-III PtdIn-3 kinase (**Figures 1B**, **2D**), indicating that *Arabidopsis* likely assembles an architecturally similar particle.

### Identification of the *vps38* T-DNA Insertion Mutants

**FIGURE 3:**
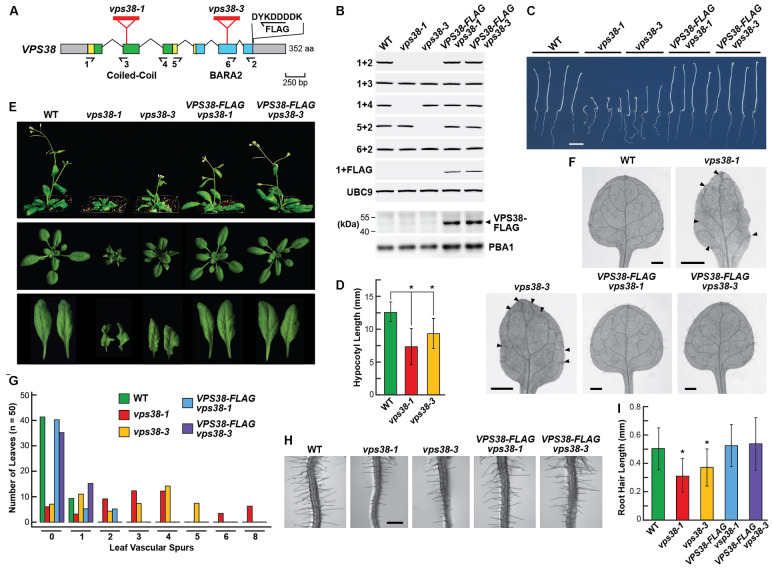
*Arabidopsis vps38* mutants display severe growth defects. **(A)** Diagram of the *Arabidopsis VPS38* gene. Colored boxes indicate coding regions and gray boxes indicate 5′- and 3′ UTRs. Coiled-coil and BARA2 domains are in green and blue, respectively. Lines identify introns. Amino acid length is indicated on the right. Red triangles locate the T-DNA insertion sites in the *vps38* mutants. Arrows show the location of the primers used for RT-PCR in **(B)**. Position and sequence of the FLAG tag appended to the VPS38-FLAG complementation construction are shown. **(B)** RT-PCR and immunoblot analysis of the *vps38* mutant and *VPS38-FLAG* complementation lines. Total RNA isolated from wild-type (WT), *vps38-1*, and *vps38-3* mutants without or with the *VPS38-FLAG* transgene were subjected to RT-PCR using primers indicated in **(A)**. RT-PCR of *UBC9* was included as a control. Presence of the transgene was verified with the 1+FLAG primer pair. Expression of the VPS38-FLAG protein was detected in total seedling extracts by immunoblot analysis with anti-FLAG antibodies. Analysis of the extracts with anti-PBA1 antibodies was used to confirm near equal protein loading. **(C)** Images of etiolated seedlings grown in the dark for 5 days, showing the shorter hypocotyl and wandering growth pattern of roots from the *vps38* mutants. Scale bar = 0.5 cm. **(D)** Hypocotyl lengths of WT and *vps38* mutants as grown in **(C)**. Each bar represents the mean (±SD) of at least 40 seedlings. Asterisks indicate significant differences from WT by Student’s *t*-test (*p* < 0.01). **(E)** Images of 5-week-old seedlings grown on soil under a LD photoperiod showing the dwarfed rosettes and curled leaves of *vps38* plants. **(F)** Abnormal vascular tissue development in *vps38* leaves. Representative primary leaves were cleared of chlorophyll to show the vascular patterns. Arrowheads indicate vascular spurs without connections for the peripheral vascular system of *vps38* leaves. Scale bar = 0.5 mm. **(G)** Quantitative distribution of vascular spurs in leaves as shown in **(F)**. Fifty leaves were quantified for each genotype. **(H)** *vps38* mutants have shorter root hairs. Shown are sections of 6-day-old roots 1.8-cm away from the root tip. Scale bar = 0.5 mm. **(I)** Quantitation of root hair length for roots shown in **(H)**. Ten roots were quantified for each genotype. Asterisks indicate significant differences from WT by Student’s *t*-test (*p*-value < 0.01).

To study the function(s) of *Arabidopsis VPS38* genetically, we searched the available germplasm collections for T-DNA mutants impacting the locus. Two mutant lines were available from the *Arabidopsis* Biological Resource Center (ABRC) which were confirmed by DNA sequence analysis; one contains a T-DNA insertion within the 2nd exon after nucleotide 507 downstream of the start codon (SAIL_552_F02), which was designated here as *vps38-1*, and a second contains a T-DNA insertion within the 5th exon after nucleotide 1,886 downstream of the translation start site (SALK_094540C), which was designated as *vps38-3* based on the allele nomenclature of Lee et al. (2018) (**Figure 3A**). Homozygous individuals were identified in the selfed F2 generation by PCR genotyping, indicating that plants missing VPS38, unlike mutants impacting other subunits of the class-III PtdIn-3 kinase complex (Fujiki et al., 2007; Qin et al., 2007; Harrison-Lowe and Olsen, 2008; Lee et al., 2008b; Xu et al., 2011; Wang et al., 2012), produce viable pollen that will germinate.

Reverse-transcribed (RT)-PCR analysis of these seedlings revealed that the full-length *VPS38* transcript failed to accumulate in the *vps38-1* and *vps38-3* plants. However, partial transcripts were evident, suggesting that shortened translation products of *VPS38* could be synthesized. If the first nascent initiator methionine codon downstream of the T-DNA at position 160 was used in *vps38-1* plants, a shorter 193-residue protein was possible that would be missing most of the coiled-coil region but include all of the BARA2 domain. By contrast, the *vps38-3* mutant could express most of the polypeptide (295 residues) beginning at the first methionine residue and include all of the coiled-coil region and much of the BARA2 domain, and thus might represent a weaker allele (**Figure 3A** and Supplementary Figure S1). In self crosses of the heterozygous *vps38* mutants, we noticed significantly reduced segregation of the mutant alleles, suggesting that VPS38 impacts reproduction. Instead of the expected 1:4 segregation ratio for homozygous mutant plants, lower ratios of 1:5.7 for *vps38-1* (*n* = 478 seeds) and 1:5.1 for *vps38-3* (*n* = 496 seeds) were obtained, using the stunted-seedling *vps38* phenotype for discrimination (see below).

### *vps38* Mutations Compromise *Arabidopsis* Growth and Reproduction

Compared to wild-type (WT) Col-0, plants homozygous for the *vps38-1* and *vps38-3* mutations displayed an array of growth and developmental defects. To confirm that these phenotypes were associated with the mutations, we generated transgenic lines in which both mutant alleles were rescued with an intronless construction assembled with a *VPS38* cDNA. It encoded the full-length protein bearing an in-frame, C-terminal FLAG tag, and was expressed under the control of the native *VPS38* promoter encompassed within a ∼2,000-bp region upstream of the translation start site. As shown in **Figure 3B**, plants homozygous for either of the *vps38-1* or *vps38-3* alleles and the *VPS38:VPS38-FLAG* transgene were absent the full-length *VPS38* transcript but now expressed the *VPS38-FLAG* transcript as judged by RT-PCR of total seedling mRNA. The complemented lines also accumulated the corresponding FLAG-tagged protein as judged by immunoblotting total seedling extracts with anti-FLAG antibodies (**Figure 3B**).

Etiolated *vps38-1* and *vps38-3* seedlings germinated well but displayed stunted hypocotyl elongation as compared to WT or the *VPS38-FLAG* complementation lines (**Figures 3C,D**). While the mutant hypocotyls grew reasonably upright, we noticed that their roots had a wandering downward growth despite near normal elongation, suggesting a reduced sensitivity to gravity. When grown in the light under a long-day (LD) photoperiod (16-h light/8-h dark), the green seedlings developed a substantially dwarfed rosette that contained smaller, wrinkly, and serrated leaves (**Figure 3E**). *vps38* cotyledons remained flat but were smaller than normal (Supplementary Figure S3A). Root hair elongation was also compromised, with the root hairs of *vps38* roots being significantly shorter than those of WT or the *VPS38-FLAG* complementation lines (**Figures 3H,I**). A slight delay in flowering in LD was observed, which was consistent with an overall delay in rosette development of the *vps38* mutants (data not shown). The shorter hypocotyls of etiolated seedlings and the dwarf phenotype and shorter root hairs of green seedlings were especially strong for the *vps38-1* line, suggesting that this allele more severely compromises VPS38 activity.

Detailed analysis of the vascular pattern in *vps38-1* and *vps38-3* leaves demonstrated that VPS38 is also important for vein development. As shown in **Figures 3F,G**, the vasculature of WT leaves or those from the *VPS38-FLAG* complementation lines were highly connected, whereas both the *vps38* lines displayed a high incidence of vascular spurs and breaks, especially when assessing the vascular bundles that ring the leaf periphery. These disconnections were uncommon in cotyledons, suggesting that the defect arises later as the true leaves emerge (Supplementary Figures S3A,B). As this leaf phenotype has been linked to impaired auxin transport and/or sensitivity (Pahari et al., 2014), we considered it possible that VPS38 influences auxin signaling.

**FIGURE 4:**
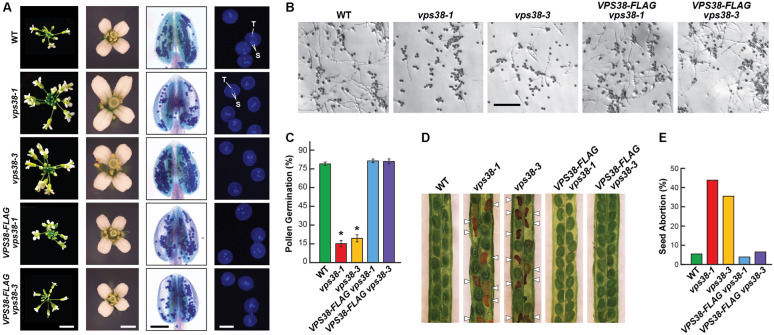
VPS38 is required for robust reproduction. **(A)** *vps38* mutants have altered inflorescence morphology but produce viable pollen. Shown are representative inflorescence apices, flowers, anthers, and pollen grains from WT, *vps38-1*, *vps38-3*, and *VPS38-FLAG* complementation plants grown for 50 days under a LD photoperiod. The anthers were incubated with Alexander stain to label viable pollen. Pollen grains were stained with DAPI to locate nuclei. T, tube nucleus; S, sperm nuclei. Scale bars = 1 cm, 1 mm, 100 μm, and 20 μm, respectively. **(B)** Germination of *vps38* pollen is depressed. Pollen grains from just opened flowers were incubated on pollen germination medium for 10 h. Scale bar = 300 μm. **(C)** Quantification of the percent germination for pollen shown in **(B)**. Each bar represents the average (±SD) of three independent analyses, each consisting of >300 pollen grains from 10 anthers. Asterisks indicate significant differences from WT by Student’s *t*-test (*p*-value < 0.01). **(D)** *vps38* mutants display enhanced seed abortion. Shown are self-fertilized siliques from homozygous *vps38-1* and *vps38-3* plants as compared to WT or the corresponding *VPS38-FLAG* complementation lines. Aborted seeds are indicated by the arrowheads. **(E)** Quantification of the abortion rate for self-fertilized siliques as shown in **(D)**. The bars represent the means of 460–902 seeds measured from 25 to 45 siliques of each genotype.

In contrast to *Arabidopsis* mutants affecting core subunits of the class-III PtdIn-3 kinase complex (Fujiki et al., 2007; Qin et al., 2007; Harrison-Lowe and Olsen, 2008; Lee et al., 2008a,b; Xu et al., 2011; Wang et al., 2012), both *vps38* mutants were fertile and successfully developed homozygous seeds. We noticed that the mutant inflorescences were generally more compact and contained more florets, and that the mutant flowers were slightly larger than WT or the *VPS38-FLAG* complementation lines (**Figure 4A**). Incubating the anthers with Alexander stain, which discriminates live from aborted pollen by uptake of the stain, showed that the *vps38* pollen have normal viability with few, if any, dead pollen grains evident (**Figure 4A**). Male gametogenesis also appeared to proceed normally based on 4,6-diamidino-2-phenylindole (DAPI) staining of pollen nuclei. Like WT and *VPS38-FLAG* complementation pollen grains, almost all the *vps38* grains collected from homozygous plants at anthesis contained an obvious tube nucleus along with two sperm nuclei (**Figure 4A**).

While prior reports indicated that *vps34*, *vps15*, and *atg6* pollen fail to germinate, *vps38-1* and *vps38-3* pollen successfully germinated but at markedly reduced numbers as compared to WT and the *VPS38-FLAG* complementation lines (**Figures 4B,C**). Slower rates of pollen tube emergence and slower rates of tube elongation were also evident. Despite these challenges, pollen development was sufficiently robust to promote female gametophyte fertilization. This slowed pollen tube growth is likely responsible for the diminished transmission of the *vps38* alleles seen in self-pollinated heterozygous *vps38* plants (see above).

Surprisingly, reproductive problems also likely extended into zygote development. Here, we observed that both *vps38-1* and *vps38-3*, but not WT or the complemented *VPS38-FLAG* lines, showed high rates of seed abortion following self-fertilization, which were easily visible in immature siliques as dark brown, shriveled seeds alongside plump green seeds that could germinate (**Figure 4D**). For the *vps38* lines, 36–45% of the seeds aborted compared to 4–8% for WT and the *VPS38-FLAG* complementation lines (**Figure 4E**). At present, we do not know which stage(s) of seed development become compromised in the mutants.

**FIGURE 5:**
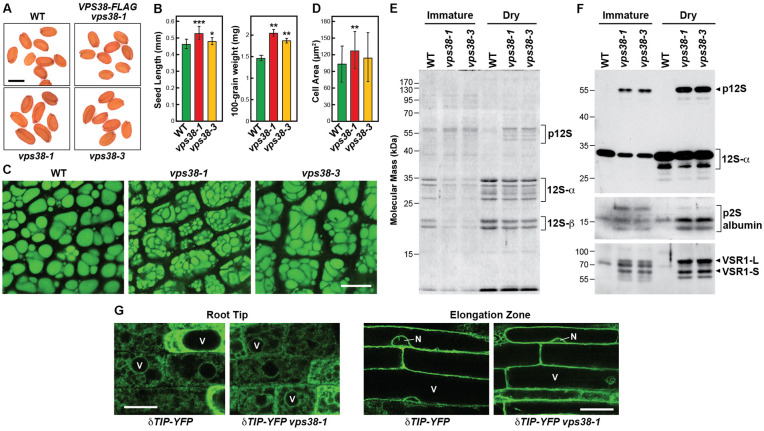
*vps38* mutants produce larger seeds and display defects in storage protein processing and vacuolar sorting receptor (VSR) accumulation. **(A,B)** *vps38* mutants produce larger seeds. **(A)** Images of seeds from WT, *vps38-1*, *vps38-3*, and *VPS38-FLAG* complementation plants. Scale bar = 0.5 mm. **(B)** Quantification of seed length and weight. Each bar represents the mean (±SD) of 60–100 seeds. Asterisks indicate significant differences from WT by Student’s *t*-test; ^∗^, ^∗∗^, and ^∗∗∗^ represent *p*-values of <0.05, <0.01, and <0.001, respectively. **(C,D)** Cotyledons of *vps38* mutants accumulate larger cells with irregularly shaped protein storage vacuoles (PSVs). **(C)** PSVs were visualized in cotyledons dissected from dry seeds by autofluorescence as detected using confocal fluorescence microscopy. Scale bar = 10 μm. **(D)** Quantification of cotyledon cell size. Each bar represents the mean (±SD) of 39–46 cells. ^∗∗^ indicates a significant difference from WT by Student’s *t*-test (*p*-value < 0.01). **(E,F)** Abnormal accumulation of 12S globulin and 2S albumin precursors, and VSR1 in *vps38* mutants. Total proteins from immature and dry seeds from WT and *vps38* mutants were either **(E)** stained for protein with Coomassie Blue, or **(F)** subjected to immunoblot analysis with anti-12S globulin, anti-2S albumin, and anti-VSR1 antibodies. Brackets/arrowhead locate the p12S precursor of the 12S-α and 12S-β globulins, and the precursor to the 2S albumins. The 80-kDa VSR1-L and 60-kDa VSR1-S versions of VSR1 are indicated by the arrowheads (Shimada et al., 2003a). **(G)** Vacuolar morphology in WT and the *vps38-1* roots expressing the tonoplast marker δTIP-YFP. Shown are images of cells either near the root tip or in the elongation zone. Scale bar = 10 μm.

Upon inspection of seeds, we also noticed that seeds produced from homozygous *vps38-1* and *vps38-3* plants were larger than WT. In fact, increases in both seed length and weight were evident, especially for the *vps38-1* allele (**Figures 5A,B**). To investigate the underpinning causes, we imaged the cotyledon cells present within mature seeds using the strong autofluorescence of their protein storage vacuoles (PSVs) (Shimada et al., 2003a). Whereas WT cotyledon cells contained numerous round PSVs, equivalent cells from the mutant cotyledons were noticeably larger and were densely packed with PSVs of irregular shapes and sizes (**Figures 5C,D**).

Aberrant PSV morphology in *vps38* seeds could have arisen from defects in vacuolar biogenesis, or problems in storage protein accumulation and/or processing. As a test of the former, we examined coalescing and mature vacuoles at the root tip and elongation zones, respectively, using the tonoplast reporter δ-TONOPLAST INTRINSIC PROTEIN (TIP) fused to YFP (Tian et al., 2004). In both zones, similar vacuolar morphologies were observed in the *vps38-1* mutant versus WT (**Figure 5G**). As a test of the latter possibility, we compared the profiles of seed storage proteins extracted from either immature or dry mature seeds. In particular, 2S albumin and 12S globulin are the major storage proteins in *Brassicaceae*, which arise from longer precursors synthesized on the rough ER (Shimada et al., 2003a). The precursors are transported by membrane trafficking to PSVs and then converted into their mature forms (α and β chains for 12S globulin and large (9 kDa) and small (7.5 kDa) forms of 2S albumin) by vacuolar proteases (Shimada et al., 2003b). Strikingly, levels the 12S precursor of globulin with an apparent molecular mass of 54 kDa was substantially higher while those for the mature 12S-α and 12S-β subunits products at 26–32 kDa and 18–20 kDa, respectively, were substantially lower in *vps38-1* and *vps38-3* seeds, as judged by subjecting total seed extracts to SDS-PAGE and either staining for protein with Coomassie blue or immunoblotting with anti-12S antibodies (**Figures 5E,F**). Substantial increases in the 2S albumin precursor were also evident in *vps38* seeds as seem by immunoblotting with anti-2S antibodies (**Figure 5F**).

These storage proteins require the vacuole sorting receptor (VSR)-1 receptor for PSV deposition through the MVB-sorting pathway (Shimada et al., 2003a; Di Sansebastiano et al., 2017). Whereas the respective long (L) and short (S) isoforms of VSR1 at 80- and 60-kDa were barely detectable in WT cotyledons, they were highly abundant in both immature and dry *vps38-1* and *vps38-3* seeds (**Figure 5F**). This strong increase implied either that VSR1 has become immune to turnover in the *vps38* backgrounds, possibly upon failure to enter PSVs or the central vacuole, or that its synthesis has dramatically increased as the *vps38* mutants attempt to restore seed storage protein deposition.

### VPS38 Promotes Autophagy

In mammals and yeast, PtdIn-3P is an important signal for autophagic membrane dynamics by interacting with several regulatory factors that bind this lipid (Pankiv et al., 2010; Olsvik et al., 2015), including the membrane-spanning ATG18 protein (WIPI1-4 in mammals) that helps deliver lipids to the growing phagophore (Dooley et al., 2014), the ATG16-binding protein ATG21 that connects the ATG12-5/ATG16 ligase with the phagophore to drive ATG8 lipidation (Juris et al., 2015), and the *Arabidopsis* SH3P2 and FREE1 proteins that promote autophagy, presumably by encouraging phagophore closure and autophagosome delivery (Zhuang et al., 2013; Gao et al., 2014, 2015). Accumulating data outside of plants show that the class-III PtdIn-3P kinase complex I containing ATG14 generates the PtdIn-3P needed for autophagy (Kihara et al., 2001; Itakura et al., 2008; Diao et al., 2015), but whether VPS38 and its class-II complex also might contribute to autophagy in plants was unknown.

**FIGURE 6:**
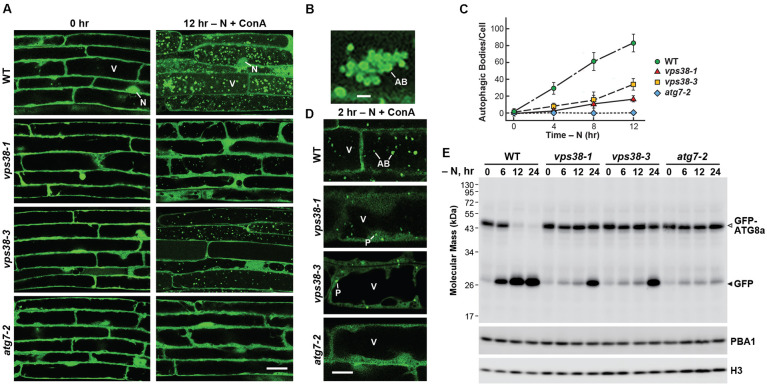
*vps38* mutants display defects in autophagy. **(A)** *vps38* mutants accumulate fewer autophagic bodies upon nitrogen starvation. Seven-day-old WT, *vps38-1*, *vps38-3*, and *atg7-2* seedlings were grown under a LD photoperiod on nitrogen-rich solid MS medium, transferred to nitrogen-deficient liquid MS medium containing 1 μM ConA, and incubated in the light for 12 h. Root cells were imaged by confocal fluorescence microscopy. Scale bar = 20 μm. V, vacuole; N, nucleus. **(B)** Close-up image of a cluster of autophagic bodies that accumulate in WT vacuoles starved for nitrogen as in **(A)**. Scale bar = 5 μm. **(C)** Time course for the accumulation of autophagic bodies upon nitrogen starvation. Roots were treated as in **(A)**. The accumulation of autophagic bodies were quantified by confocal microscopy and expressed as a mean (±SD) of at least 25 cells. **(D)** *vps38* mutants accumulate GFP-ATG8a-decorated autophagic structures in the cytoplasm but poorly assemble autophagic bodies. The seedlings were grown as in **(A)** but incubated in nitrogen-deficient medium containing 1 μM ConA for only 2 h. Scale bar = 5 μm. AB, autophagic body. P, possible phagophore/autophagosome. V, vacuole. **(E)** *vps38* mutants display defects in autophagic transport as judged by the release of free GFP from GFP-ATG8a (Chung et al., 2010). Seedlings were grown under continuous light on nitrogen-rich MS liquid medium, transferred to nitrogen-deficient liquid MS medium, and then harvested at the indicated times. Total seedling extracts were immunoblotted with anti-GFP antibodies, using anti-PBA1 and anti-histone H3 antibodies to confirm near equal protein loading. Open and closed arrowheads locate the GFP-ATG8a fusion and free GFP, respectively.

To test if VPS38 regulates autophagy, we introgressed the *vps38-1* and *vps38-3* mutations into an *Arabidopsis* background expressing the autophagic reporter GFP-ATG8a (Thompson et al., 2005), and examined the dynamics of autophagic vesicles by confocal fluorescence microscopy. Here, the seedlings were exposed to nitrogen stress to induce autophagy and treated with the vacuolar ATPase inhibitor concanamycin A (ConA) to stabilize autophagic bodies and thus enhance their detection (Thompson et al., 2005). Surprisingly, we found that VPS38, presumably through its assembly of PtdIn-3P, also promoted autophagy. Similar to previous studies (Thompson et al., 2005; Chung et al., 2010; Suttangkakul et al., 2011), GFP-ATG8a-decorated autophagic bodies accumulated to high levels in the vacuoles of WT root cells when starved for nitrogen (**Figure 6A**). In fact, sufficiently high numbers of autophagic bodies collected that large vesicle clusters could be observed after prolonged nitrogen deprivation (12 h) (**Figure 6B**). This accumulation was markedly reduced in both the *vps38-1* and *vps38-3* lines, but not to the same extent as that seen with the strong autophagy mutant *atg7-2* (Chung et al., 2010), indicating that VPS38 is important but not essential for the transport of autophagic cargo to the vacuole (**Figures 6A,C**).

Interestingly, when we inspected fluorescent images of root cells soon after the onset of starvation (2 h), we found that the *vps38* mutants appeared to be stalled at an intermediate stage of autophagic vesicle assembly. Whereas, WT root cells accumulated both vacuolar autophagic bodies and cytoplasmic autophagic structures reminiscent of phagophores [and possibly autophagosomes (Le Bars et al., 2014)], and *atg7-2* cells accumulated neither, the *vps38* mutants only accumulated the cytoplasmic structures (**Figure 6D**).

For another quantitative view of VPS38-regulated autophagy, we exploited observations that the GFP moiety becomes detached from GFP-ATG8a upon its autophagy-mediated deposition into *Arabidopsis* vacuoles, and accumulates in a free form because of its resistance to vacuolar proteases (Chung et al., 2010; Li et al., 2014; Marshall et al., 2015). For WT *Arabidopsis* expressing GFP-ATG8a, this transport was seen by the appearance of free GFP following exposure of the seedlings to nitrogen starvation (**Figure 6E**). Breakdown was easily evident within 6 h and most GFP-ATG8a was cleaved by 24 h in WT plants, which by contrast was mostly blocked in *atg7-2* plants (**Figure 6E**). The *vps38-1* and *vps38-3* seedlings displayed an intermediate response. While little free GFP accumulated after 6–12 h of nitrogen starvation, strong accumulation of this cleavage product was seen by 24 h. Together with the dampened accumulation of autophagic bodies, we concluded that VPS38 influences autophagic transport in plants, although not as strongly as components within the core autophagic machinery such as ATG7.

To identify which aspects of the autophagic system might be impacted by VPS38, we examined by immunoblotting the levels of the ATG12-ATG5 conjugate needed for ATG8 lipidation, and the levels of the final ATG8-PE adducts. Here, the identity of the ATG12-ATG5 conjugate in WT plants was confirmed by immunoblot detection of a ∼50-kDa form of ATG5 with anti-ATG5 antibodies, which was replaced by the expected 39-kDa non-conjugated form in the *atg7-2* background that blocks assembly of this adduct, and by the absence of both forms in *atg5-1* plants (Thompson et al., 2005). ATG8-PE adducts assembled from the nine ATG8 isoforms expressed in *Arabidopsis* (Doelling et al., 2002) were visualized following their separation from free ATG8 by SDS-PAGE in the presence of 6 M urea, with the identities of the lipidated products then verified by their sensitivity to phospholipase D (Chung et al., 2010).

**FIGURE 7:**
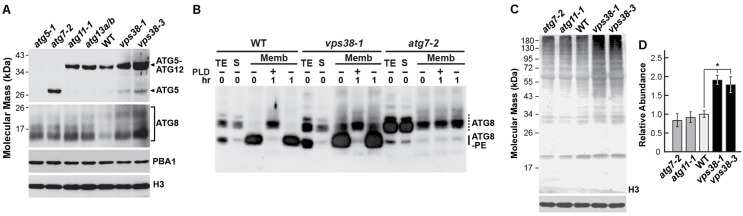
*vps38* mutants accumulate normal levels of the ATG12-ATG5 and ATG8-PE adducts but have elevated levels of ATG8 and ubiquitin conjugates. **(A)** *vps38* mutants assemble the ATG12-ATG5 adduct normally. Total seedling extracts from 14-day-old homozygous WT, *atg5-1*, *atg7-2*, *atg11-1*, *atg13a-2/b-2*, *vps38-1*, and *vps38-3* seedlings grown under a LD photoperiod on nitrogen-rich solid MS medium were subjected to immunoblot analysis with anti-ATG5 and ATG8a antibodies. Free ATG5 and the ATG12-ATG5 adduct are indicated by the arrowheads. Immunoblots with anti-PBA1 and anti-histone H3 antibodies were included to confirm near equal protein loading. **(B)** *vps38-1* plants accumulate lipidated ATG8 normally. WT, *vps38-1*, and *atg7-2* seedlings were grown on N-rich liquid medium for 7 days and then exposed to N-deficient medium for 2 days before extraction. Total seedling extracts (TE) were separated into the soluble (S) and membrane (Memb) fractions by centrifugation. The membrane fraction was solubilized in Triton X-100 and incubated with or without phospholipase D (PLD) for 1 h. Samples were then subjected to SDS-PAGE in the presence of 6 M urea and immunoblotted with antibodies against ATG8a. Dashed lines locate free ATG8. Solid lines locate ATG8-PE adducts. **(C)** *vps38* mutants accumulate more ubiquitin-protein conjugates as compared to WT and the *atg7-2* and *atg11-1* autophagy mutants. Total seedling extracts from 14-day-old seedlings grown on nitrogen-rich solid MS medium were subjected to immunoblot analysis with anti-ubiquitin antibodies. An immunoblot with anti-histone H3 antibodies was included to confirm near equal protein loading. **(D)** Quantification of ubiquitin signals in **(D)**. The immunoblot signals were quantified from scanned images, normalized using the signal from anti-histone H3 antibodies, and expressed relative that found for WT seedlings. Each bar represents the mean of three independent experiments. Asterisk indicates significant differences from the WT according to a Student’s *t*-test (*p*-value < 0.05).

Unexpectedly, the *vsp38* mutations had little impact on the assembly of either conjugate. Like WT and autophagy mutants impacting ATG11 and ATG13 that work outside of these conjugation events (Li and Vierstra, 2012; Marshall and Vierstra, 2018), the *vps38-1* and *vps38-3* lines effectively synthesized the ATG12-ATG5 adduct with little free ATG5 evident (**Figure 7A**). The *vps38-1* mutant, like WT, also accumulated high levels of the ATG8-PE adduct in the membrane fraction, which was not seen upon fractionation of *atg7-2* extracts (**Figure 7B**). However, the involvement of VPS38 in autophagy more generally was evident in the immunoblots by the higher levels of total ATG8 (both free and lipidated forms) in the *vps38* mutants (**Figures 7A,B**), which is a hallmark of dampened autophagic turnover (Chung et al., 2010). This response was likely underpinned by up-regulated expression of *ATG8* genes when the pathway is compromised (Sláviková et al., 2005; Thompson et al., 2005), along with suppressed autophagic turnover of the ATG8 protein, as seen when other components of the *Arabidopsis atg* system are eliminated (Chung et al., 2010; Suttangkakul et al., 2011).

Because ubiquitylation has been connected to autophagy through its regulation of endosome dynamics and the use of ubiquitin to mark autophagic cargo for recognition by various ubiquitin-binding receptors (Scheuring et al., 2012; Marshall and Vierstra, 2018), we examined the profile of ubiquitin conjugates in *vps38* plants. As compared to WT, *atg7-2*, and *atg11-1* seedlings, the *vsp38-1* and *vps38-3* seedlings accumulated significantly more ubiquitylated species (**Figures 7C,D**). The fact that these increases were higher than that seen with *atg7-2* seedlings argues that some of these VPS38-affected ubiquitylated targets are not involved in autophagy but participate in other aspects of protein trafficking, including TGN and MVB dynamics, that also employ ubiquitylation events (Gerth et al., 2017; Noack and Jaillais, 2017).

**FIGURE 8:**
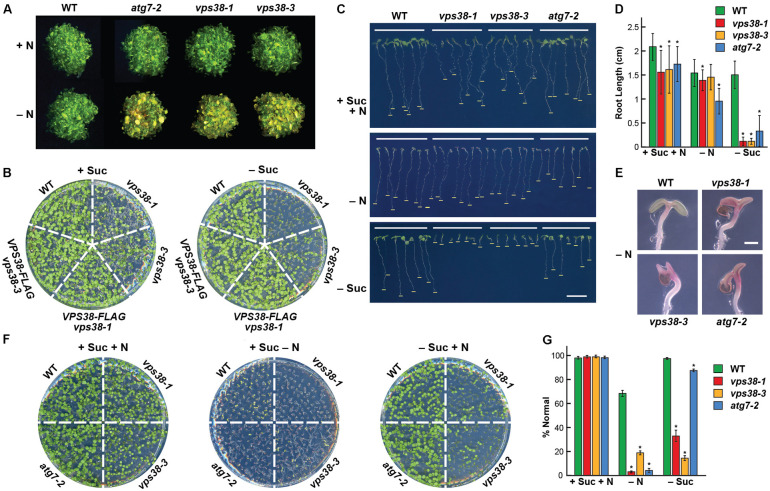
*vps38* mutants have increased sensitivity to nitrogen and fixed-carbon starvation. **(A)** Enhanced sensitivity to nitrogen starvation. WT, *vps38-1*, and *vps38-3* plants were grown on nitrogen-containing liquid MS medium for 1 week and then transferred to either nitrogen-rich (+N) or nitrogen-deficient (–N) liquid medium for 1 week. WT and autophagy-defective *atg7-2* seedlings were included as negative and positive controls, respectively. **(B)** Enhanced sensitivity to fixed-carbon starvation. WT, *vps38-1*, *vps38-3*, and the *VPS38* complementation seedlings were grown on either sucrose-containing (+Suc) or sucrose-deficient (–Suc) solid MS medium under a LD photoperiod for 2 weeks. **(C)** *vps38* roots are hypersensitive to fixed-carbon starvation relative to *atg7-2* roots. Seedlings were grown vertically for 10 days on solid MS medium with or without nitrogen (–N) and sucrose (–Suc). Bars indicate the extent of root growth. **(D)** Quantitation of the effects of fixed-carbon and nitrogen starvation on root growth as shown in **(C)**. Each bar represents the average of 37–60 seedlings (±SD). Asterisks indicate significant differences by the Student’s *t*-test (*p*-value < 0.01). **(E)** Close-up views of cotyledons showing the enhanced anthocyanin accumulation in *vps38* and *atg7-2* seedlings under nitrogen starvation. Scale bar = 0.5 mm. **(F)** *vps38* shoots are hypersensitive to fixed-carbon starvation relative to the autophagy mutant *atg7-2*. Seedlings were grown horizontally for 10 days on solid MS medium with or without nitrogen (–N) and sucrose (–Suc). **(G)** Quantitation of the effects of fixed-carbon and nitrogen starvation based on the survival of seedlings shown in **(F)**. Percent normal assessed whether the plants did not accumulate high levels of anthocyanins when grown without nitrogen, or did not displayed severely stunted development when grown with or without sucrose, as compared to WT plants grown in replete medium. Each bar represents the average of 37–60 seedlings (±SD). Asterisks indicate significant differences by the Student’s *t*-test (*p*-value < 0.01).

For further proof that *vps38* mutations impair autophagy, we examined the sensitivity of *Arabidopsis* seedlings to nitrogen and fixed-carbon starvation, which require a functional autophagy system for robust survival (Doelling et al., 2002; Hanaoka et al., 2002; Chung et al., 2010; Li et al., 2014). Like *atg7-2* plants, the *vps38-1* and *vps38-3* plants grew slower and became more chlorotic when grown on nitrogen-free liquid medium (**Figure 8A**). A hypersensitivity to fixed-carbon starvation was also seen. When grown in the light under a LD photoperiod in the presence of sucrose, the *vps38* plants were already smaller than WT or the *VPS38-FLAG* complementation lines, in agreement with the importance of VPS38 for normal seedling growth (see **Figure 3E**). However, when placed on sucrose-free medium, the *vps38* seedlings strongly arrested growth even in the light, while WT and the *VPS38-FLAG* complementation seedlings remained relatively healthy and continued growing (**Figure 8B**). Again, the *vps38-1* allele was noticeably more sensitive relative to the *vps38-3* allele (**Figures 8A,B**).

To assess how the starvation responses of *vps38* plants compare to those of the strong autophagy mutant *atg7-2*, we grew the mutants side-by-side under a LD photoperiod on growth medium with and without nitrogen or sucrose (**Figures 8C–G**). In replete medium (+N+Suc), root elongation and shoot development were noticeably slower for the *vps38-1* and *vps38-3* lines, like the *atg7-2* mutant. When placed on nitrogen-deficient medium, growth of the *vps38-1* and *vps38-3* seedlings was further impaired to now more closely resemble *atg7-2* seedlings. Root growth was depressed (albeit slightly more for the *atg7-2* line), and the cotyledons displayed strongly reduced unfolding and expansion, and accumulated high concentrations of anthocyanins, which is a hallmark of nitrogen-stress (**Figures 8C–E**).

Strikingly, when the plants were grown on medium missing sucrose, the *vps38* seedlings were hypersensitive as compared to both wild type and *atg7-2* seedlings. Root growth almost completely stalled for the *vps38-1* and *vps38-3* mutants (**Figures 8C,D**). Shoot growth was also markedly suppressed in both *vps38* mutants, whereas growth of the *atg7-2* mutant was nearly identical to that of WT (**Figures 8F,G**). Together, we discovered that plants missing VPS38 are more sensitive to the lack of fixed-carbon provided by the growth medium that one would expect for a strong autophagy mutant even under lighting that encouraged cotyledon development and photosynthesis. This additional sensitivity implies that VPS38 influences other processes beyond autophagy during fixed-carbon deprivation.

### VPS38 Impacts Endosomal Trafficking

**FIGURE 9:**
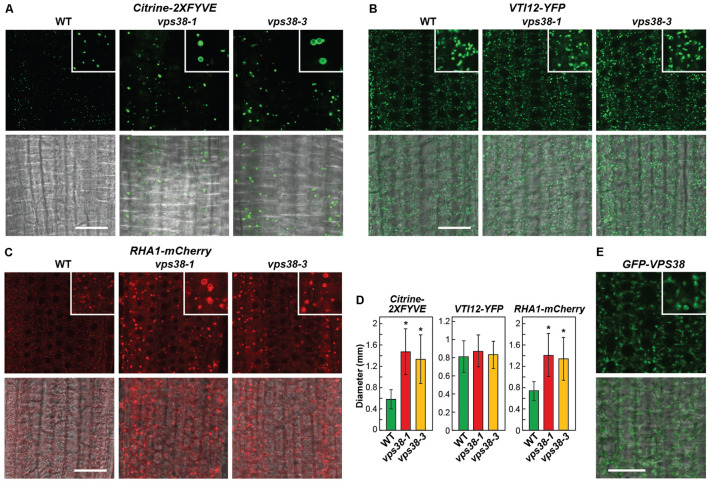
VPS38 influences late endosome morphology. Each reporter in **(A–C)** was introgressed into *vps38-1* and *vps38-3* plants and grown on nitrogen-rich solid MS medium under a LD photoperiod. Root tip cells from 7-day-old seedlings were imaged in all panels by confocal fluorescence microscopy. Shown are fluorescence (top) and merged bright field and fluorescence images (bottom). The inset is a 3× magnification. Scale bars = 20 μm. **(A)** *vps38* mutants accumulate larger PtdIn-3P-decorated vesicles detected by the Citrine-2XFYVE reporter that specifically binds to PtdIn-3P. **(B)** *vps38* mutants accumulate phenotypically normal vesicles decorated with the early endosome marker VTI12-YFP that encodes a member of the v-SNARE complex critical for docking and/or fusion of transport vesicles within the *trans*-Golgi network (TGN). **(C)** *vps38* mutants accumulate larger vesicles decorated with the MVB/late endosome marker RHA1-mCherry that encodes an *Arabidopsis* homolog of RAB5 which regulates late endocytosis. **(D)** Quantification of the vesicle sizes detected in **(A–C)**. Vesicle diameters were measured from the microscopic images using NIH-ImageJ. Each bar represents the mean of >100 vesicles measured from at least five different seedlings. Asterisks indicate significant differences from the WT according to a Student’s *t*-test (*p*-value < 0.01). **(E)** VPS38 localizes to the endomembrane system in *Arabidopsis*. GFP-VPS38 was expressed in WT roots grown for 1 week on nitrogen-rich solid MS medium under a LD photoperiod. Shown are fluorescence (top) and merged bright field and fluorescence images (bottom). The inset is a 3× magnification. Scale bar = 20 μm.

PtdIn-3P has also been implicated in endomembrane sorting through the TGN, particularly for events involving MVBs, late endosomes, and ultimately vesicle fusion to the tonoplast membrane delineating vacuoles (Gerth et al., 2017; Noack and Jaillais, 2017). To support a role for VPS38 in this procession, we analyzed the *vps38-1* and *vps38-3* mutants by confocal fluorescence microscopy using reporters that follow sections of this transport system. The Citrine-2XFYVE reporter harbors two PtdIn-3P-binding FYVE domains from human Hepatocyte Growth Factor-Regulated Tyrosine Kinase Substrate (HGS/Hrs), thus enabling it to serve as a biosensor of PtdIn-3P (Simon et al., 2014). As shown in **Figures 9A,D**, Citrine-2XFYVE was detected in cytoplasmic puncta whose numbers did not increase but each was enlarged in the *vps38* backgrounds. Often rings were seen in the focal planes, suggestive of dilated MVBs. VSP TEN-INTERACTING (VTI)12 protein is a v-SNARE located on the TGN and early endosomes (Sanmartin et al., 2007). Here, no differences in size or density for VTI12-decorated vesicles were evident when comparing *vps38-1* and *vps38-3* to WT (**Figures 9B,D**). Finally, RHA1 is a Rab5 GTPase homolog that follows vacuolar trafficking of soluble protein cargo through MVBs and late endosomes (Sohn et al., 2003). The mCherry derivative of RHA1 labeled vesicles that did not increase in number in the *vps38* backgrounds but were substantially enlarged like those labeled with Citrine-2XFYVE, suggesting that VPS38 restricts the size of MVBs/late endosomes decorated with PtdIn-3P (**Figures 9C,D**).

And finally, we examined the localization of VPS38 itself using a GFP-tagged version expressed in WT roots via the cauliflower mosaic virus *35S* promoter. Confocal fluorescence images detected GFP-VPS38 in bright cytoplasm foci of ∼1 μm often at the center of spreading trails (**Figure 9E**), which were reminiscent of the endomembrane system labeled with VTI12-YFP (**Figure 9B**). An intriguing possibility is that these foci represent phagophores emerging from the endomembrane system and undergoing decoration with PtdIn-3P. Taken together, while VPS38 has been reported to influence early endosome and TGN trafficking in animals (Liang et al., 2008), our studies are more consistent with the scenario that the product of its associated complex – PtdIn-3P influences MVBs/late endosome trafficking in plants.

### Gravitropism Is Impaired in *vps38* Mutants

**FIGURE 10:**
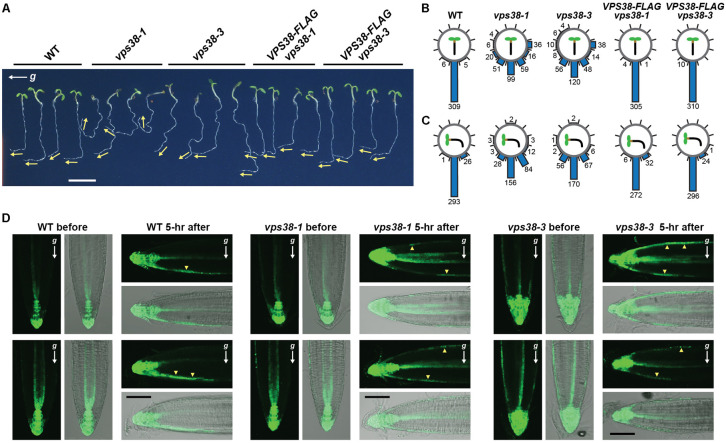
VPS38 is essential for robust gravitropism and the gravity-mediated re-distribution of auxin. **(A)** *vps38* mutants have a dampened gravitropism response. WT, *vps38-1*, *vps38-3*, and the *VPS38-FLAG* complementation lines were grown on nitrogen-rich solid MS medium in a LD photoperiod. After 5 days, the plates were rotated 90° from vertical and imaged 1 day afterwards. Arrows indicate the new growth direction of the roots. Scale bar = 0.5 cm. **(B,C)** Quantification of root orientation toward gravity before **(B)** and 1 day after the 90° rotation **(C)**. Each circle contains measurements from 291 to 320 roots with the angles of each sub-divided into 30° increments. The total number in each sub-division is indicated. **(D)** *vps38* roots have a dampened auxin redistribution during gravitropism, as measured by the auxin-responsive *DR5:GFP* reporter. Five-day-old seedlings were imaged by fluorescence microscopy either before or 5 h after a 90° reorientation of the seedlings from vertical. Shown are pairs of root tips for each genotype imaged either by fluorescence (top) and merged bright field and fluorescence (bottom). Arrowheads locate GFP signals on the upper and lower sides of the roots. The gravity vector (*g*) is shown by the arrows. Scale bar = 100 μm.

The meandering, wavy growth of *vps38* roots (see **Figure 3C**), along with the report that the PtdIn-3 kinase inhibitors LY294002 and wortmannin impair *Arabidopsis* gravitropism (Joo et al., 2005; Jaillais et al., 2006; Alvarez et al., 2016), implicated VPS38 in gravity perception. As shown in **Figure 10B**, quantitation of this meander without reorienting the plants easily showed that *vps38-1* and *vps38-3* roots display a weaker gravity response as compared to WT or the *VPS38-FLAG* complementation lines. For further support, we grew seedlings on a vertical agar surface for 7 days, and then rotated the surface 90° to assay for gravitropic reorientation of root growth 1 day later. Whereas WT and *VPS38-FLAG* complementation roots changed their growth direction toward the new gravity vector, the *vps38* mutants were significantly less effective (**Figures 10A,C**).

Given that the gravity-triggered reorientation of root growth is driven by asymmetric auxin transport from the root tip up toward the shoot (Friml, 2003), we assessed auxin flow in *vps38* roots using the auxin-sensitive reporter *DR5:GFP*, whose expression is induced by the hormone (Ottenschläger et al., 2003). As observed previously (Zhang et al., 2010), auxin concentrations were high in the root tip and extended up the central stele and columella cell files when WT roots were held in the vertical position (**Figure 10D**). This distribution changed when the roots were tilted horizontally to now include flow along the downward cortical flank close to the epidermis (**Figure 10D**). This new auxin stream preferentially inhibits cell expansion on the downward flank to eventually adjust root growth back toward vertical (Friml, 2003). *vps38-1* and *vps38-3* roots similarly retained flow of auxin from the root tip into the vascular tissue for roots held vertical. However, once reoriented to horizontal, auxin flow in the *vps38* roots, as detected by *DR5:GFP* expression, was seen in both the downward and upward peripheral flanks (**Figure 10D**), which in turn should lessen asymmetric auxin flow and dampen subsequent gravitropism.

To test if phototropism was similar influenced by VPS38, we assayed the reorientation of *vps38* seedlings to unilateral light. Here, 5-day-old etiolated seedlings were exposed to white light from the side and measured over a 30-h time sequence for their hypocotyl curvature toward the light. Whereas WT seedlings seedlings strongly reoriented, with most seedlings eventually growing directly toward the light, the *vps38-1* and *vp38-3* seedlings displayed a sluggish response (Supplementary Figures 4A,B). However, when correcting for the slowed elongation of *vps38* hypocotyls (Schöller et al., 2018) (see **Figure 3C**), the phototropic bending rate of the mutants largely overlapped with that of WT over this time frame, implying that phototropism does not strickly depend on VPS38.

### Mis-Localization of PIN Proteins in *vps38* Cells

**FIGURE 11:**
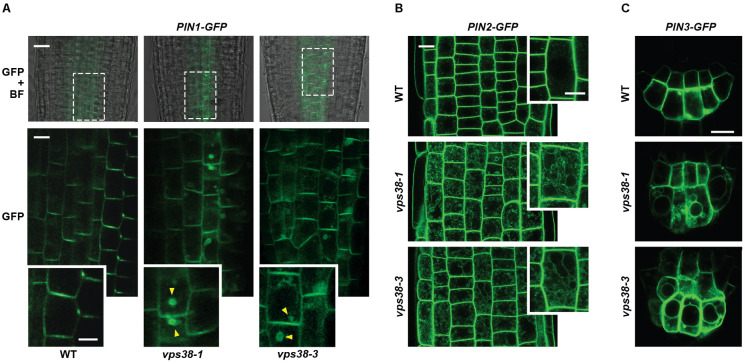
VPS38 regulates the distribution of the PIN1, PIN-2, and PIN-3 auxin efflux carriers. The PIN-GFP reporters were introgressed into the *vps38-1* and *vps38-3* backgrounds and grown on nitrogen-rich solid MS medium under a LD photoperiod. Root tip cells from 7-day-old seedlings were imaged by confocal fluorescence microscopy. Insets show magnifications of individual cells. The *vps38* mutants show abnormal reticulated distributions of the PIN reporters within the cytoplasm in addition to their plasma membrane locations. **(A)** PIN1-GFP localization. Shown are merged bright field and fluorescence images (top) and fluorescence images (bottom) at the core of the root just distal to the root tip (defined by the boxes in the merged images). Twofold enlargements of representative cells are below each image. Possible BFA bodies containing PIN1-GFP are indicated by the arrowheads. Scale bar = 20 μm. **(B)** PIN2-GFP localization. Shown are fluorescence images just distal to the root tip. Twofold enlargements of representative cells are shown on the right side of each image. Scale bar = 15 μm. **(C)** PIN3-GFP localization. Shown are cells at the root tip. Scale bar = 20 μm.

The directional flow of auxin is accomplished in part by a family of PIN-FORMED (PIN) auxin efflux carriers whose polar distribution on the plasma membrane, driven by both endosome recycling and vacuolar degradation, encourages asymmetric auxin flow (Kleine-Vehn et al., 2008; Adamowski and Friml, 2015). To examine whether changes in the cellular distributions of PIN carriers underpinned the dampened gravitropism of *vps38* mutants, we analyzed their cellular locations by confocal fluorescence microscopy of lines expressing GFP-tagged versions of PIN1, PIN2, and PIN3 from their native promoters (**Figure 11**), which represent the main PIN proteins expressed in roots (Adamowski and Friml, 2015). Importantly, the cellular distributions of all three reporters were altered in the *vps38-1* and *vsp38-3* backgrounds. In WT roots, PIN1-GFP was concentrated in the stele and columella files, particularly on the downward flank of the plasma membrane of each cell (**Figure 11A**). However, for *PIN1-GFP vps38* root cells, a reticulated pattern within the cytoplasm was also seen in addition to the plasma membrane concentration, suggesting that PIN1-GFP was partitioning to a greater extent into the endosomal trafficking system (**Figure 11A**). In support, many *PIN1-GFP vps38* cells contained large cytoplasmic puncta resembling brefeldin-A (BFA) bodies, which are thought represent intermediate locations of PIN proteins as they dynamically flow into and out of the plasma membrane (Kleine-Vehn et al., 2008).

Likewise, the distribution of PIN2-GFP and PIN3-GFP, which are abundant in root cortical tissue and root apex, respectively (Adamowski and Friml, 2015), displayed similar reticulated patterns in the cytoplasm in *vps38* roots, which were not evident in WT roots (**Figures 11B,C**). The relocation of PIN3-GFP was especially striking with the GFP signal obvious both within the cytoplasm and on the vacuolar surface of the mutants in addition to its plasma membrane distribution.

To further support the position of PIN2 with the endomembrane trafficking system, we visualized by confocal fluorescence microscopy the PIN2-GFP reporter in parallel with the fluorescent probe FM4-64, which is widely used as an endocytic tracer (Rigal et al., 2015). In WT root cells, FM4-64 rapidly moved within 6 h from the plasma membrane to now label the endomembrane system (Supplementary Figure S5). Signal from PIN2-GFP did not co-localize with these internal vesicles as most of its fluorescent signal remained on the plasma membrane. However, in *vps38-1* and *vps38-3* roots, internal signals for PIN2-GFP appeared whose patterns matched those of FM4-64 (Supplementary Figure S5), strongly suggesting that PIN2 increases its residency within the endomembrane system without VPS38. Taken together, our analyses revealed that VPS38 influences vesicular trafficking, leading to defects in PIN protein localization and subsequent asymmetric auxin flow necessary for robust root gravitropism.

## Discussion

It has long been appreciated that PtdIn-3P, and by inference its associated kinases, are important for eukaryotic membrane identity and vesicular trafficking (Balla, 2013), but its exact roles in plants have remained largely enigmatic due to the lack of informative genetic tools for the sole known class-III PtdIn-3 kinase complex that generates this lipid (Gerth et al., 2017; Noack and Jaillais, 2017). For example, while the PtdIn-3 kinase-selective inhibitors wortmannin and LY294002 have tentatively connected PtdIn-3P to various events (Takatsuka et al., 2004; daSilva et al., 2005; Joo et al., 2005; Jaillais et al., 2006; Takáč et al., 2013; Fujimoto et al., 2015; Alvarez et al., 2016), the essential nature of the single *Arabidopsis* genes that encode the core VPS34, VPS15, and ATG6 (VPS30/Beclin1) subunits has stalled further investigations beyond its importance to pollen germination (Fujiki et al., 2007; Qin et al., 2007; Harrison-Lowe and Olsen, 2008; Lee et al., 2008a,b; Xu et al., 2011; Wang et al., 2012).

We, and a recent study by Lee et al. (2018), overcame this hurdle by examining mutants eliminating the accessory subunit VPS38, which exchanges with ATG14 to distinguish the complex-I and complex-II isoforms (Kihara et al., 2001; Itakura et al., 2008). Although amino acid sequence similarities between VPS38 from *Arabidopsis* and other plants and their yeast and mammalian orthologs are low, both the predicted presence of the signature BARA2 and coiled-coil domains in the plant sequences (Rostislavleva et al., 2015), and our demonstration by several interaction assays that *Arabidopsis* VPS38 interacts with the VPS34, VPS15, and ATG6 subunits of the core class-III complex confirmed its identity. The only notable difference was the absence of the lipid-binding C2 domain in most of the plant versions, which could reflect a unique way that the plant VPS38 polypeptides interact with membranes.

Strikingly, homozygous *Arabidopsis vps38* mutants are viable but display a range of defects consistent with aberrant endosomal sorting, vacuolar trafficking, and autophagy (this report; Lee et al., 2018), which are consistent with studies with yeast and mammalian cells that demonstrated the importance of PtdIn-3P to these processes (Kihara et al., 2001; Itakura et al., 2008). In our study, the defects were confirmed by a pair of *vps38* alleles, along with their rescue by a *VPS38-FLAG* transgene. In side-by-side phenotypic comparisons of the two *vps38* mutants, the *vps38-3* allele consistently elicited weaker effects, suggesting that the predicted truncation of the vps38-3 protein missing part of the BARA2 domain accumulates and retains modest activity. Phenotypic abnormalities were evident for hypocotyl growth, leaf development, vascular patterning, and root hair elongation. Most notably, while floral morphology and pollen development proceeded normally, pollen grain germination was dampened and the resulting homozygous seeds aborted with increased frequency, implying that complications arise during the later stages of male gametogenesis and subsequent embryogenesis. However, these challenges were not sufficiently strong to block generation of viable progeny from self-fertilized homozygous *vps38* plants.

Given that a functional ATG14 remains in *vps38* plants, we presume that the class-III PtdIn-3 kinase complex I assembled with ATG14 provides the necessary PtdIn-3P to maintain plant cell viability in the absence of VPS38. In support, Lee et al. (2018) analyzed the profile of PtdIns in the *vps38-1* and *vsp38-2* backgrounds and found no changes in PtdIn-3P abundance and only slight increases in PtdIn-4P and bis-phosphorylated PtdIn-4,5P_2_ forms. Clearly, studies of triple mutants missing VPS38, ATG14a, and ATG14b will be necessary to address the essential nature of this subunit in the class-III complex. Our expectation is that *vps38 atg14a atg14b* triple mutants will display male gametogenesis defects similar to the *vps34*, *vps15*, and *atg6* null mutants (Fujiki et al., 2007; Qin et al., 2007; Harrison-Lowe and Olsen, 2008; Lee et al., 2008a,b; Xu et al., 2011; Wang et al., 2012).

Studies on several membrane trafficking events supported roles for VPS38 and its associated class-III PtdIn-3 kinase complex. As seen with mutants affecting several other components of the plant endosomal sorting pathways [e.g., VPS29, SNX1, and FREE1 (Shimada et al., 2006; Gao et al., 2015; Reguera et al., 2015)], the *vps38* lines displayed defects in sorting seed storage proteins to PSVs, resulting in the accumulation of non-processed precursors for several storage proteins and a hyper-accumulation of the protein storage receptor VSR1 that helps shuttle cargo into PSVs (Shimada et al., 2003a; daSilva et al., 2005). The end results were slightly larger seeds that contained larger cotyledon cells filled with abnormally shaped PSVs. Likewise, the shape and vein patterning of *vps38* leaves strongly resembled those missing UNHINGED, the *Arabidopsis* homolog of VPS51 that localizes to the TGN and pre-vacuolar compartments to direct retrograde sorting (Pahari et al., 2014).

Whereas Vps38/UVRAG was not directly connected to autophagy in yeast and mammalian cells, with ATG14 fulfilling this role in decorating the autophagic membranes with PtdIn-3P (Kihara et al., 2001; Itakura et al., 2008), VPS38 did have a significant influence in *Arabidopsis*. Using the GFP-ATG8a reporter to track autophagy (Thompson et al., 2005; Chung et al., 2010), we found that *vps38* seedlings accumulate fewer autophagic bodies under nitrogen starvation and display reduced rates of deposition of this reporter into vacuoles as measured by the autophagy-mediated release of free GFP from the fusion. The impacts of the *vps38* mutations were not as strong as those affecting core autophagy components [e.g., *atg7-2* (Chung et al., 2010)], implying that the PtdIn-3P needed to decorate autophagic membranes is either not essential, or more likely that this lipid is also synthesized by the class-III PtdIn-3 kinase complex I assembled with ATG14. Intriguingly, while the accumulation of autophagic bodies was suppressed in *vps38* root cells subjected to nitrogen starvation, we observed the rapid appearance of cytoplasmic puncta that could represent phagophores/autophagosomes stalled in assembly and/or prevented from fusing with vacuole.

Presumably, VPS38 (and the rest of the PtdIn-3 kinase complex) generates PtdIn-3P that helps recruit various PtdIn-3P-binding proteins needed to assemble autophagosomes and deliver them to the vacuole [e.g., FREE1 and SH3P2 (Zhuang et al., 2013; Gao et al., 2014, 2015)]. Assays of the ATG12-ATG5 and ATG8-PE adducts suggest that the effects of VPS38 are downstream of these two conjugation events. This was unanticipated for ATG8-PE given that the ATG12/ATG5/ATG16 ligase complex responsible for this essential lipidation step is recruited to autophagic membranes in other eukaryotes, at least in part, by PtdIn-3P (Juris et al., 2015).

The stunted, chlorotic phenotype seen here for *vps38* plants starved for nitrogen were similar to those impacting other components of the *atg* system, albeit weaker than mutants impacting core components (Hanaoka et al., 2002; Thompson et al., 2005; Suttangkakul et al., 2011; Li et al., 2014). However, the response to fixed-carbon starvation induced by removing sucrose from the growth medium but still keeping the plants illuminated, was dramatically different. Whereas *atg7-2* seedlings continued to grow like WT without this external carbon source, root elongation and expansion of cotyledons and leaves for both the *vps38-1* and *vps38-3* seedlings were strongly arrested. A similar hypersensitivity to sucrose deprivation has also been reported for *Arabidopsis* mutants impacting VPS29, SNX1, and GRAVITROPISM DEFECTIVE (GRV)-2 required for late steps in endocytosis (Tamura et al., 2007; Kleine-Vehn et al., 2008; Silady et al., 2008), suggesting that this starvation response is more connected to defects in late endosomal sorting more generally, rather than to autophagy specifically.

In other organisms, PtdIn-3P plays a key role in vesicle trafficking, especially at steps involving MVBs, late endosomes, and final deposition into vacuoles/lysosomes (Balla, 2013; Di Sansebastiano et al., 2017). Localization studies by us and Lee et al. (2018) support such a role in plants. While the VTI12-YFP marker for early endosomes (Sanmartin et al., 2007) did not show a change in vesicle number or size in the *vps38* mutants, substantially dilated vesicles were detected using the late endosome marker RHA1-GFP (Sohn et al., 2003). This dilation was also seen with the Citrine-2XFYVE biosensor of PtdIn-3P (Simon et al., 2014), suggesting that these swollen vesicles are decorated with this PtdIn. Presumably, this binding arises from PtdIn-3P derived from the remaining active class-III PtdIn-3 kinase complex assembled with ATG14a/b. However, puncta morphologically similar to those seen in WT roots expressing RHA1-mCherry and Citrine-2XFYVE were seen in roots expressing GFP-VPS38, strongly suggesting that VPS38 also associates with the late endosome fraction. Clearly co-localization studies among the various endosome markers, ATG14a/b, and VPS38 are now needed to determine if the complex-I or -II isoforms have distinct or overlapping intracellular destinations.

Our discovery that *vps38* mutants have compromised gravitropism provides further support that the class-III PtdIn-3 kinase, and by inference PtdIn-3P, dramatically influences the trafficking of proteins among the plasma membrane, endomembrane system, and vacuoles (Kleine-Vehn et al., 2008; Gerth et al., 2017; Noack and Jaillais, 2017). The gravitropism defect was caused, at least in part, by diminished differential auxin flow along the upward and downward flanks of reoriented roots and was likely underpinned by changes in PIN protein cycling between the plasma membrane and internal vesicles and/or vacuoles, and not likely by altered vacuolar biogenesis. In fact, a common theme of our localization studies with PIN1-GFP, PIN2-GFP, and PIN3-GFP was the presence of the reporters in internal membranes and vesicles in *vps38* roots, which were not evident in WT roots. For PIN3-GFP, we could even detect the reporter seemingly associated with the tonoplast in *vps38-1* root tips, further strengthening the proposal that the PIN proteins are degraded by autophagy (Kleine-Vehn et al., 2008). We hypothesize that these internal pools of PIN proteins reflect dampened (as opposed to accelerated) cycling of these efflux carriers between the plasma membrane and internal membrane stores, and/or slowed delivery to the vacuole for breakdown.

Ultimately, adjustments of PIN protein polarity necessary for driving asymmetric auxin flow are compromised in the *vps38* backgrounds leading to weaker growth responses toward gravity. In a similar fashion, mutations in the GRV2 protein important to vacuolar sorting also display dampened gravitropism (Tamura et al., 2007; Silady et al., 2008). When connected with the prior observation that UNHINGED, which localizes to the TGN and prevacuolar compartments and regulates vesicle movements, also impacts auxin signaling (Pahari et al., 2014) and that the *zig-1* gravitropism mutant encoding the VTI11 SNARE protein is hypersensitive to wortmannin (Alvarez et al., 2016), the links between PtdIn-3P, membrane trafficking, auxins, and root gravitropism become more obvious. Conversely, loss of VPS38 had only a slight effect on hypocotyl phototropism, but this dampened light response was complicated by the slowed hypocotyl elongation of the mutants.

In sum, this study provides detailed genetic analyses of PtdIn-3P synthesis in plants through the study of class-III PtdIn-3 kinase complex II assembled with VPS38. While homozygous mutants were viable, in contrast to mutants eliminating the core VPS34, VPS15, and ATG6 (VPS30/Beclin1) subunits, a surprisingly large number of phenotypes were seen here related to pollen germination, leaf and vascular morphology, seed development, sensitivity to nitrogen and fixed-carbon deprivation, and gravitropism. Much of these defects appear connected to challenges in endomembrane dynamics and vacuolar transport, indicating that this kinase complex and its PtdIn-3P product have pleiotropic functions in directing intracellular protein trafficking and autophagy. Further studies of *vps38* mutants in combination with *atg14a/b* mutants impacting the complex-I isoform of the class-III PtdIn-3 kinase should eventually reveal the full importance of this PtdIn in plant growth and development.

## Materials and Methods

### Sequence and Phylogenetic Analysis of VPS38

The *A. thaliana VPS38* gene was identified in the Col-0 ecotype genomic sequence by BLASTP and TBLASTN from National Center for Biotechnology Information (NCBI) server^3^, using the coding regions from the yeast *Vps38* and human *UVRAG* genes as queries. Reciprocal BLASTP analysis was performed with the GenBank DNA database to identify possible *VPS38/UVRAG* orthologs in other eukaryotes. For phylogenetic analyses, related VPS38 protein sequences were aligned by EMBL-ESI Multiple Alignment using Fast Fourier Transform version 7 under the default settings, and with the relative ATG14 as an outgroup. The alignment was used to generate an Unweighted Pair Group Method with Arithmetic Mean tree with MEGA7 (Kumar et al., 2016) via 1000 bootstrap replications, which was shown in TreeView^4^. Identical and similar amino acids were visualized by BoxShade^5^, and protein domains were predicted by PFAM^6^. A possible three-dimensional model of *Arabidopsis* VPS38 was generated by SWISS-MODEL^7^, using the structure of yeast Vps38 [PDB entry 5DFZ (Rostislavleva et al., 2015)] as the template, and was visualized in PyMOL v1.8^8^.

### Genome and Transcriptional Analysis

Transcript abundance for loci encoding subunits of the class-III PtdIn-3 kinase complex were assessed in GENEVESTIGATOR^9^ and displayed by a *Z*-score heatmap generated in R version 3.3.2 using hierarchical clustering. Genomic PCR for mapping the T-DNA insertion sites used total DNA isolated from 7-day-old seedlings. RT-PCR analysis began with total RNA isolated using the RNeasy mini kit (QIAGEN) from 14-day-old seedlings grown on Murashige and Skoog (MS) medium (Sigma-Aldrich). RNA was converted to cDNA via the SuperScript III first-strand synthesis system (Invitrogen) with oligo(dT)20 primers, and then used as templates for RT-PCR with gene-specific primers. See Supplementary Table S1 for the list of oligonucleotide primers used in this study.

### Protein/Protein Interaction Assays

Standard GAL4-based Y2H assays based on *HIS3* expression were performed as described (Gingerich et al., 2007) with slight modifications. cDNAs encoding the full protein sequence of *VPS38*, *ATG6*, *VPS15*, and *VPS34* were generated by RT-PCR of total seedling RNA from WT *A. thaliana* Col-0 and subsequently cloned into the pDONR221 entry plasmid by Gateway BP reactions. These fragments were then recombined in-frame into the pDEST22 plasmid harboring the *GAL4* activation domain and into the pDEST32 plasmid harboring the *GAL4* DNA-binding domain via Gateway LR reaction (Thermo Fischer Scientific). Plasmids were co-transformed pairwise into yeast strain MaV203 as recommended (Thermo Fischer Scientific), and assayed for protein-protein interactions by 2-day growth at 30°C on synthetic complete medium lacking histidine, leucine and tryptophan, and containing 25 mM 3-amino-1,2,4-triazole, using synthetic complete medium lacking leucine, and tryptophan as the control.

For Y2H assays using the split ubiquitin Protein A/LexA/VP16 (PLV) system, positive interactions were based on association of the N-terminal and C-terminal fragments of ubiquitin (NubG and Cub, respectively), which allows proteolytic release and nuclear import of the chimeric PLV transcription factor to then activate *HIS3* expression (Stagljar et al., 1998). The cDNAs were introduced into pMETYCgate/pNXgate32/pXNgate22-based plasmids system (Chen et al., 2012) by Gateway recombination. Baits were fused to the N-terminus of Cub-PLV and transformed into yeast strain THY.AP4 (mating type a), while candidate preys were fused to either the N-terminus or C-terminus of NubG and transformed to yeast strain THY.AP5 (mating type α). After mating, the diploid THY.AP4 THY.AP5 cells were placed on synthetic complete medium lacking histidine, leucine, tryptophan, uracil, and adenine to test for protein-protein interactions by growth. The NubG and NubWT empty vectors were used as negative and positive controls, respectively.

For BiFC assays, the entry plasmids described above were recombined into the pSITE-nEYFP-C1 vector (ABRC CD3-1648) containing YFP N-terminal fragment 1-174 (nYFP), and the pSITE-cEYFP-C1 vector (ABRC CD3-1649) containing YFP C-terminal fragment 175-239 (cYFP) (Martin et al., 2009; Li et al., 2014). The nYFP and cYFP fusions were transiently co-expressed in leaves from 5-week-old *Nicotiana benthamiana* via *Agrobacterium tumefaciens*-mediated infiltration as modified (Grimsley et al., 1986; Marshall et al., 2015). Reconstituted YFP fluorescence was assayed after 24–48 h by confocal laser scanning microscopy of the infiltrated leaves.

### Plant Materials

The *vps38-1* (SAIL_552_F02), and *vps38-3* (SALK_094540C) T-DNA insertion mutants (Sessions et al., 2002; Alonso et al., 2003) in the *A. thaliana* Col-0 ecotype background were obtained from *Arabidopsis* Biological Resource Center (ABRC, Ohio State University). The mutants were backcrossed twice to WT Col-0 and then selfed to obtain homozygous seed. The T-DNA insertion sites were confirmed by genomic PCR using a T-DNA left border-specific primer in combination with a gene-specific primer (see Supplementary Table S1). Homozygous *atg5-1*, *atg7-2*, *atg11-1*, and *atg13a-1 atg13b-2*, mutants, and plants harboring *35S:GFP-ATG8a* in Col-0 background were described previously (Thompson et al., 2005; Chung et al., 2010; Suttangkakul et al., 2011; Li and Vierstra, 2014). The *DR5:GFP* auxin-responsive reporter line (Ottenschläger et al., 2003), lines expressing the *PIN1:PIN1-GFP*, *PIN2:PIN2-GFP*, and *PIN3:PIN3-GFP* reporters (Benková et al., 2003; Abas et al., 2006; Zhang et al., 2013), and lines expressing the endosomal markers RHA1-mCherry, VTI12-YFP, and Citrine-2XFYVE (Sohn et al., 2003; Sanmartin et al., 2007; Simon et al., 2014) were as described. Average sizes of the vesicles detected by these reporters were quantified by NIH-ImageJ^10^. The *Arabidopsis* line expressing *δTIP-YFP* were as described (Tian et al., 2004). All the reporters were introgressed into the *vps38* mutants by genetic crosses; homozygous offspring were selected by RT-PCR and/or fluorescence from the F3 backcross generation.

For *vps38* complementation studies, each *VPS38* transgene under the control of its native promoter was constructed from the full-length coding region introduced into the pMDC43 plasmid (Curtis and Grossniklaus, 2003), which was digested with *Hind*III and *Asc*I to remove the CaMV *35S* promoter. The native promoter (-2,000 bp fragment ending at the initiator methionine codon) was PCR amplified from *A. thaliana* Col-0 genomic DNA and fused into pMDC43 backbone by Gibson Assembly (New England BioLabs) to construct the pMDC43-*pVPS38* vector. Codons for the FLAG tag (DYKDDDDK) was appended in-frame to the 3′ end by PCR with an appropriate oligonucleotide, and the amplified *VPS38-FLAG* fragment was cloned into the pMDC43-*pVPS38* plasmid via Gateway BP/LR reaction described above. The *GFP-VPS38* transgene expressed under the control of the CaMV *35S* promoter was created by inserting the full-length coding region of *VPS38* in between the *35S* promoter-*GFP* coding region and *NOS* 3′ UTR sequences in the pMDC43 plasmid using Gateway LR reactions. The *VPS38:VPS38-FLAG* and *35S:GFP-VPS38* constructions were transformed into *vps38* mutant and WT plants, respectively, via the *Agrobacterium*-mediated, floral-dip method.

### Plant Phenotypic Assays

Seeds were surface-sterilized, sown in culture dishes containing full-strength MS medium containing 1% sucrose, 1.4% agar, and 2 mM MES-KOH (pH 5.7), and then exposed to continuous white light for 12 h at 4°C to induce uniform germination. Effects of *VPS38* on hypocotyl growth were measured from seedlings maintained at 24°C either in the dark or under a LD photoperiod of white light (16-h light/8-h dark) for 5 days. Hypocotyls lengths were measured by scanned images by NIH-ImageJ. For nitrogen starvation, surface-sterilized seeds were germinated and grown for 1 week at 23°C in liquid MS medium under continuous white light irradiation (Suttangkakul et al., 2011). The medium was then substituted with MS medium missing nitrogen for an additional week. For fixed-carbon starvation, seeds were sown on solid MS medium missing sucrose and containing 1% agar, and grown under a LD photoperiod of white light for 1 week.

Vascular tissue patterning was performed as described (Pahari et al., 2014). Briefly, 14-day-old cotyledons and 21-day-old first leaves were cleared of chlorophyll with ethanol:acetic acid (3:1) for 3 h, transferred to 70% ethanol for 1 h, and then incubated overnight in 95% ethanol. The cotyledons and leaves were viewed with a light microscope to detect disconnections in vascular tissue.

For gravitropism assays of roots, the seedlings were grown at 21°C under a LD photoperiod of white light on solid MS medium plates that were held vertically. After 1 week, the plates were rotated 90° and imaged 24 h later. Angles between the direction of gravity and the growth directions of the roots were measured by NIH-ImageJ. Gravitropic responses of hypocotyls were measured by the same method, except that the plates were incubated in darkness for 3 days before reorientation. For the effects of gravity on the distribution of the *DR5:GFP* reporter, confocal fluorescence microscopic images of 5-day-old seedlings were captured 5 h after the 90° rotation. The phototropic responses of hypocotyls were examined in 5-day-old seedlings grown in the dark on solid MS medium plates held vertically. The plates were then illuminated on one side with 100 μmol m^-2^ s^-1^ white light for various times before measuring the hypocotyl angle from vertical. Correcting the *vps38* mutants for slowed hypocotyl elongation used the normalization method of Schöller et al. (2018).

### Pollen Viability Assays

To test for pollen viability, anthers dissected from freshly opened flowers were incubated for several hour with Alexander’s stain [1 ml 1% malachite green in 95% ethanol, 5 ml 1% acid fuchsin in water, 0.5 ml 1% orange G in water, 10 ml ethanol, 25 ml glycerol, 5 g phenol, 5 g chloral hydrate, and 4 ml glacial acetic acid, all dissolved in 50 ml water (Alexander, 1969)]. Pollen germination was examined after a 10-h incubation of fresh pollen in solid medium containing 0.01% boric acid, 1 mM CaCl_2_, 1 mM Ca(NO_3_)_2_, 1 mM MgSO_4_, 18% sucrose, and 0.5% agar, pH 7 (Lee et al., 2008b). Pollen nuclei were identified by fluorescence microscopic examination of pollen grains under UV light after staining with DAPI.

### Microscopy and Image Analysis

Fluorescence cell images were obtained with a Nikon A1Si laser scanning confocal microscope, using 358-nm light combined with a 461-nm filter for DAPI, 488-nm light combined with the 500–550-nm filters for the GFP, Citrine, and YFP images and seed autofluorescence, 543-nm light combined with 565–615-nm filters for the mCherry images, and 515-nm light combined with the 640-nm filters for the FM4-64 images. Fluorescence images were processed using the NIS-Elements software (Nikon) and converted to TIF files. Cellular dynamics of the PIN-GFP reporters combined with FM4-64 were monitored by confocal fluorescence microscopy of 7-day-old seedlings.

### Immunoblot Analyses

Levels of ATG8, ATG5, and the ATG12-ATG5 conjugate, GFP-ATG8a, VPS38-FLAG, and ubiquitin conjugates were measured by immunoblot analysis of total seedling extracts prepared by homogenizing 100 mg of seedlings in 200 μL of 2X SDS-PAGE sample buffer [100 mM Tris-HCl (pH 6.8), 4% SDS, 0.2% bromophenol blue, and 20% glycerol], heating the samples to 100°C for 2 min, and then clarifying the extracts by brief centrifugation. Antibodies against *Arabidopsis* ATG5, ATG8a, PBA1, and plant Ub were as described (Smalle et al., 2002; Thompson et al., 2005; Kim et al., 2013). Anti-histone H3 antibodies was provided by Abcam (product number AB1791). Monoclonal antibodies against GFP and FLAG were purchased from Roche Diagnostics (product number 11814460001) and Sigma Aldrich (product number F1804), respectively. Goat anti-rabbit- or anti-mouse secondary antibodies conjugated to horseradish peroxidase or alkaline phosphatase were obtained from KPL. Blots were developed using either the SuperSignal West Pico Chemiluminescent Substrate or the SuperSignal West Femto Maximum Sensitivity Substrate (Thermo Scientific), according to the manufacturer’s instructions. Levels of ubiquitin conjugates were quantified by scanning the immunoblots with NIH-ImageJ, using several exposures to ensure that the signals were within the linear range of the film.

Lipidation of ATG8a was monitored according to Chung et al. (2010). The total membrane fraction was collected from crude protein extracts by a 10 min centrifugation at 100,000 × *g*. The pellet was solubilized in Triton X-100, clarified, and the resulting supernatant was incubated for 1 h at 37°C without or with the addition of *Streptomyces chromofuscus* phospholipase D (Enzo Life Sciences). Samples were subjected to SDS-PAGE in the presence of 6 M urea and immunoblotted with anti-ATG8a antibodies. For the GFP-ATG8a cleavage assays (Chung et al., 2010), seedlings were grown under continuous white light on nitrogen-containing liquid MS medium before transferring the seedlings to MS medium without nitrogen. Immunoblots were performed on total seedling extracts with anti-GFP antibodies as above.

Seed protein purification and immunoblot assays were performed essentially as described (Shimada et al., 2003a; Reguera et al., 2015). Briefly, dry seeds and hydrated seeds (60 each) were homogenized in 150 μL of 2X SDS-PAGE sample buffer, subjected to SDS-PAGE, and either strained for protein with Coomassie blue or immunoblotted with anti-*Arabidopsis* 2S albumin, anti-*Arabidopsis* 12S globulin α-subunit, and anti-VSR antibodies (Reguera et al., 2015). Raw image files for the immunoblots displayed in **Figures 3**, **5**–**7** are available in Supplementary Figure S6.

## Author Contributions

FL and RDV proposed the research, analyzed the data, and wrote the paper with advice from WH. FL developed the mutant and transgenic lines and conducted most experiments. WH helped with the genetics and the transcriptome analyses.

## Conflict of Interest Statement

The authors declare that the research was conducted in the absence of any commercial or financial relationships that could be construed as a potential conflict of interest.

## Footnotes

1 https://www.genevestigator.com

2 http://bar.utoronto.ca/efp2/Arabidopsis/Arabidopsis_eFPBrowser2.html

3 https://blast.ncbi.nlm.nih.gov/Blast.cgi

4 http://taxonomy.zoology.gla.ac.uk/rod/treeview/

5 http://www.ch.embnet.org/software/BOX_form.html

6 http://pfam.xfam.org

7 http://swissmodel.expasy.org

8 http://www.pymol.org

9 https://www.genevestigator.com

10 https://imagej.nih.gov/ij/
