# Corrigendum: The Vacuolar Protein Sorting-38 Subunit of the *Arabidopsis* Phosphatidylinositol-3-Kinase Complex Plays Critical Roles in Autophagy, Endosome Sorting, and Gravitropism

**DOI:** 10.3389/fpls.2020.00261

**Published:** 2020-02-28

**Authors:** Fen Liu, Weiming Hu, Richard D. Vierstra

**Affiliations:** ^1^Department of Biology, Washington University in St. Louis, St. Louis, MO, United States; ^2^South China Botanical Garden, Chinese Academy of Sciences, Guangzhou, China

**Keywords:** *Arabidopsis*, phosphatidylinositol 3-phosphate (PtdIn-3P), vesicle trafficking, pollen, autophagy, gravitropism, polar auxin transport

In the original article, there was a mistake in Panel C of Figure 2. *Arabidopsis* VPS38 Interacts with Other Subunits of Class-III PtdIns-3 Kinase Complex as published. BiFC fluorescence images for several control plasmids were inadvertently switched with others. The corrected Figure 2 appears below.

**Figure 2:**
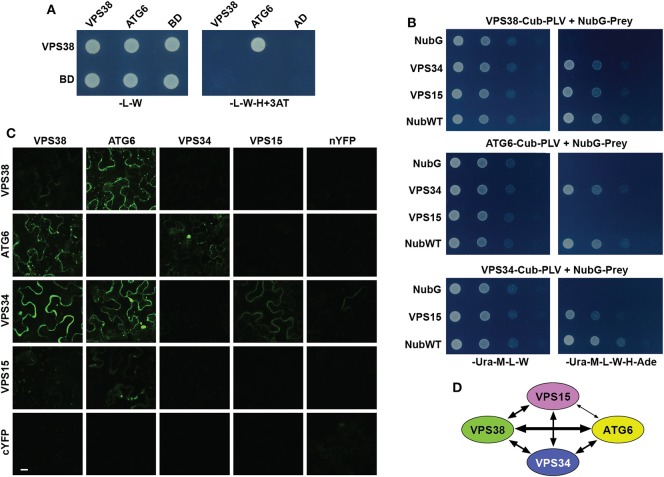
*Arabidopsis* VPS38 interacts with other subunits of class-III PtdIns-3 kinase complex. **(A)** Pairwise Y2H assays using the GAL4-based system showing that VPS38 interacts with ATG6. VSPS38 fused to the N terminus of the DNA-binding domain (BD) and ATG6 fused to the N terminus of the activation domain (AD) were co-expressed in yeast and tested for binding by growth on synthetic complete medium lacking leucine, tryptophan, and histidine (-L-W-H), and containing 3-amino-1,2,4-triazole (+3AT). Viability of the cells was confirmed by growth on medium lacking leucine and tryptophan (-L-W). **(B)** Pairwise Y2H assays by the split-ubiquitin mating system showing interactions among VPS38, ATG6, VPS15, and VPS34. Each full-length protein was expressed as a fusion to either Cub-PLV as bait or NubG as prey, and co-expressed in diploid yeast cells. Positive interactions were determined by growth of twofold serial dilutions on synthetic complete medium lacking uracil, methionine, leucine, tryptophan, histidine, and adenine (-Ura-M-L-W-H-Ade). The empty NubG and NubWT vectors were used as negative and positive controls, respectively. Viability of the cells was confirmed by growth on synthetic complete medium lacking uracil, methionine, leucine, and tryptophan (-Ura-M-L-W). **(C)** Pairwise BiFC assays showing the interactions among VPS38, ATG6, VPS15, and VPS34 *in planta*. Each full-length protein was expressed as a fusion to either N-terminal fragment (nYFP) or C-terminal fragment (cYFP) of YFP and then transiently co-expressed in *N. benthamiana* leaf epidermal cells. Appearance of the fluorescent signals was observed by confocal microscopic analysis 36 h after infiltration. Scale bar = 20 μm. **(D)** Schematic of the interactions detected among VPS38, ATG6, VPS15, and VPS34. The arrow thickness is an estimate of binding strength based on all interaction assays. The solid and dashed lines indicate interactions that were demonstrated by both Y2H and BiFC, or by just one of the methods, respectively.

The authors apologize for this error and state that this does not change the scientific conclusions of the article in any way. The original article has been updated.

