# Supplementary material for: The Vacuolar Protein Sorting-38 Subunit of the Arabidopsis Phosphatidylinositol-3-Kinase Complex Plays Critical Roles in Autophagy, Endosome Sorting, and Gravitropism
Source: Front Plant Sci. 2018 Jun 18;9:781. doi: 10.3389/fpls.2018.00781 (PMC6016017; doi:10.3389/fpls.2018.00781)
Supplement: Supplementary file 1 [file Image_1.pdf]

SUPPLEMENTAL DATA

The Vacuolar Protein Sorting-38 Subunit from the *Arabidopsis* Phosphatidylinositol-3-Kinase Complex Plays Critical Roles in Autophagy, Endosome Sorting, and Gravitropism.

Fen Liu, Weiming Hu, and Richard D. Vierstra

Supplemental Table 1.
Nucleotide Sequences of the Oligonucleotide Primers Used in This Study.

**T-DNA Left Border Primers**	
LB1 for SAIL Lines	GCCTTTTCAGAAATGGATAAATAGCCTTGCTTCC
LB1.3 for SALK Lines	ATTTTGCCGATTTCGGAAC
***VPS38* Genotyping Primers**	
vps38-1 LP	CTAATCCGATCTCTCGGTTCC
vps38-1 RP	TCTCCTTCTAGTTGGCAGTTTG
vps38-2 LP	TAAGCTTTCGTTTATCGTCGG
vps38-2 RP	CAAAGATGAGCTTCTTCACCG
***VPS38* RT-PCR Primers**	
Primer 1	ATGGAAAGAGTATCAGAGCGAAG
Primer 2	GGAGATTGGATGATTCGAATTAGCTCC
Primer 3	CAATGATTCAGTACTAACCTG
Primer 4	GAGATACCATGAACTGCTGCC
Primer 5	GGAATCATTTCCTGGTGGCAG
Primer 6	GCCTATGCTGTCTTCTTACTAAAC
FLAG/R	CCATGGCTATTTATCATCATCATCTTTGTAATCTCC
***UBC9* Primers**	
AtUBC9_F3	CCGTTGCGGAAGACATGTTTCATT
AtUBC9_R2	TAGGGCTCTTCCTTAAGGACAGTA
**Primers for constructions of complementation study vectors**	
VPS38p-Gibson-f	CGACGGCCAGTGCCATGACCAACAACTCTTCTCTAGCCTCA
VPS38p-Gibson-r	CAAACTTGTTTGATAGCTTGGCAACAATTATCTCGGAACCCACG
VPS38-pDONR221-f	GGGGACAAGTTTGTACAAAAAAGCAGGCTTAATGGAAAGAGTATCAGAGCGAAGTTTG
VPS38-pDONR221-FLAG-r	ATCATCATCTTTGTAATCTCCTCCTCCTAAATAATCGATATAATCTGGAG
VPS38-pDONR221-FLAG-r0	GGGGACCACTTTGTACAAGAAAGCTGGGTTTCATTTATCATCATCATCTTTGTAATCTC
**Primers used to generate full-length cDNAs for construction of entry vectors**	
VPS38-pDONR221-f	GGGGACAAGTTTGTACAAAAAAGCAGGCTTAATGGAAAGAGTATCAGAGCGAAGTTTG
VPS38-pDONR221-r	GGGGACCACTTTGTACAAGAAAGCTGGGTTTCATAAATAATCGATATAAT
VPS38-pDONR221-r-no-stop	GGGGACCACTTTGTACAAGAAAGCTGGGTTTAAATAATCGATATAATCTGGAG
ATG6-pDONR221-f	GGGGACAAGTTTGTACAAAAAAGCAGGCTTAATGAGGAAAGAGGAGATTCCAG
ATG6-pDONR221-r	GGGGACCACTTTGTACAAGAAAGCTGGGTTCTAAGTTTTTTTACATGAAGGC
ATG6-pDONR221-r-no-stop	GGGGACCACTTTGTACAAGAAAGCTGGGTTAGTTTTTTTACATGAAGGC
VPS15-pDONR221-f	GGGGACAAGTTTGTACAAAAAAGCAGGCTTAATGGGAAACAAAATCGCTCGTAC
VPS15-pDONR221-r	GGGGACCACTTTGTACAAGAAAGCTGGGTTTTACTTCCAGACCTTTATGGCTC

VPS15-pDONR221-r-no-stop	GGGGACCACTTTGTACAAGAAAGCTGGGTTCTTCCAGACCTTTATGGCTC
VPS34-pDONR221-f	GGGGACAAGTTTGTACAAAAAAGCAGGCTTAATGGGTGCGAACGAGTTTCGTTTC
VPS34-pDONR221-r	GGGGACCACTTTGTACAAGAAAGCTGGGTTTCAACGCCAGTATTGAGCCCATC
VPS34-pDONR221-r-no-stop	GGGGACCACTTTGTACAAGAAAGCTGGGTTACGCCAGTATTGAGCCCATC

Supplementary Figure 1. Liu *et al*.

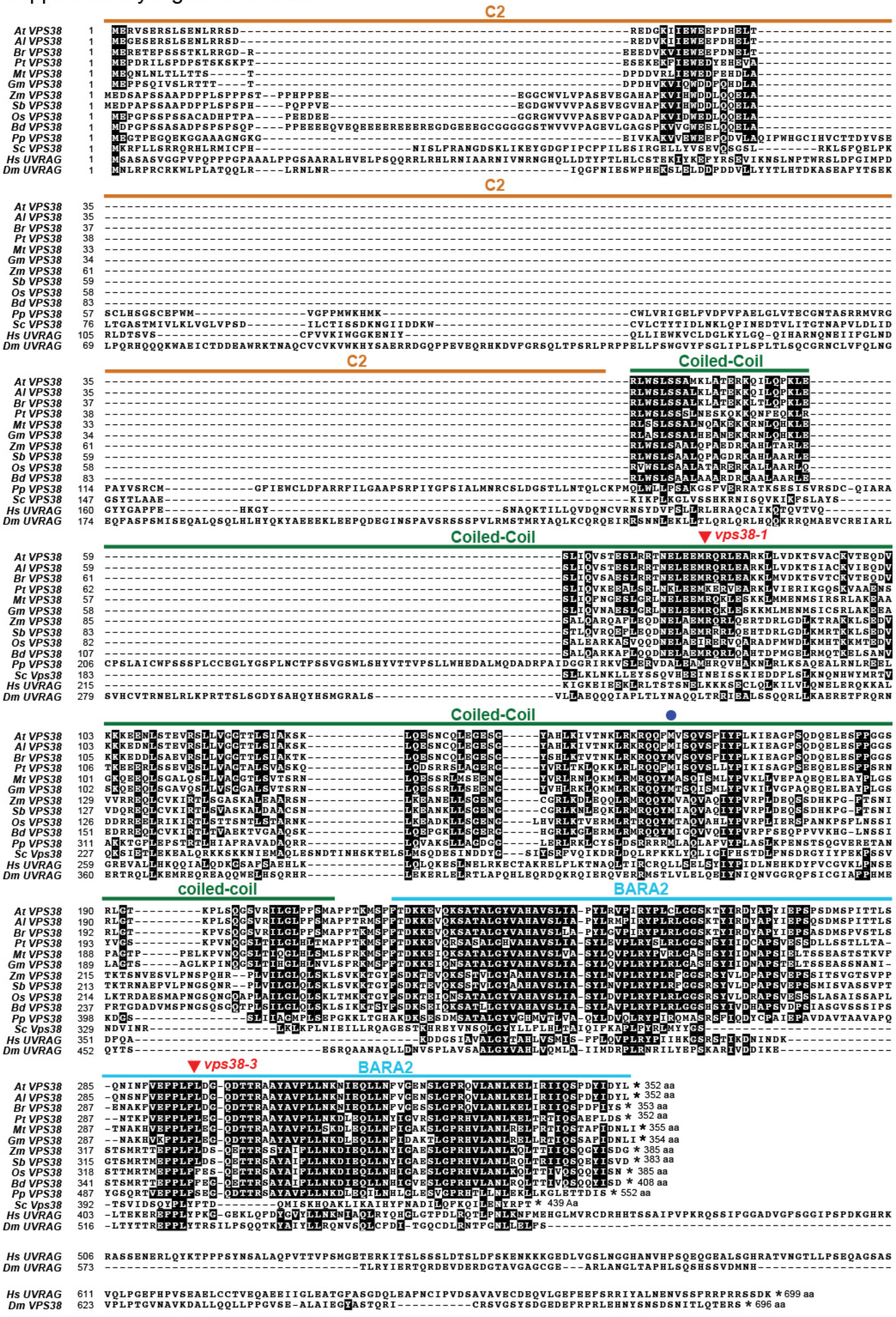

Supplemental Figure 1. Amino Acid Sequence Alignment of VPS38-Related Proteins in Various Plant, Animal, and Fungal Species.

The full protein sequences were aligned by ClustalX and visualized in BoxShade. Identical/similar amino acids are shown in black and gray boxes, respectively. The C2, coiled coil and BARA2 domains are located by the orange, green and blue lines, respectively. The amino acid length of each protein is indicated at the end of each sequence. The red arrowheads locate the insertion position of the *vps38-1* and *vps38-3* T-DNA sequences. The predicted initiator methionine for a possible vps38-1 protein is indicated by the blue circle. Asterisks denote stop codon at the end of the translated region. Species abbreviations and GenBank accession numbers are: *Al*, *Arabidopsis lyrata* (XP_020884320.1); *At*, *Arabidopsis thaliana* (AT2G32760); *Bd*, *Brachypodium distachyon* (XP_010238781.1); *Br*, *Brassica rapa* (XP_009141274.1); *Dm*, *Drosophila melanogaster* (NP_609632.1); *Gm*, *Glycine max* (XP_003531229.1); *Hs*, *Homo sapiens* (NP_003360.2); *Mt*, *Medicago truncatula* (XP_003629676.1); *Os*, *Oryza sativa* (EEC81310.1); *Pp*, *Physcomitrella patens* (XP_001779983.1); *Pt*, *Populus trichocarpa* (XP_002323989.2); *Sb*, *Sorghum bicolor* (XP_002441742.1); *Sc*, *Saccharomyces cerevisiae* (EDV08672.1); *and Zm*, *Zea mays* (ACG36492.1).

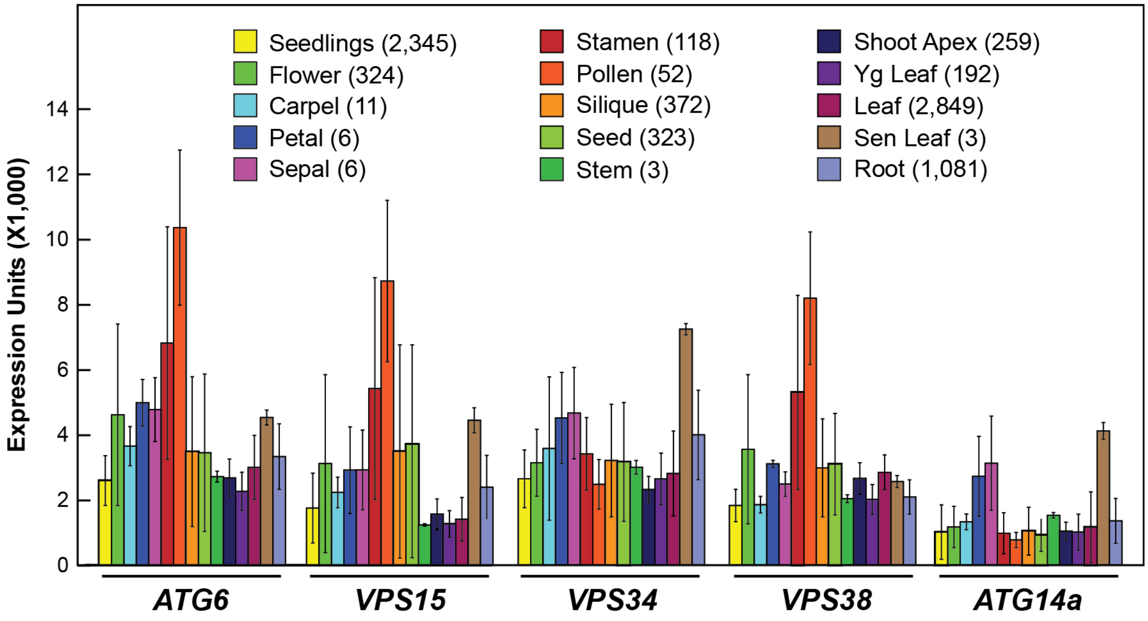

Supplemental Figure 2. Tissue Distribution of Expression for the *Arabidopsis* Genes Encoding the ATG6, VPS15, VPS34, VPS38, and ATG14a Subunits of the Class-III PtdIn-3 Kinase Complex.

mRNA abundances were obtained from GENVESTIGATOR database and expressed as relative and unit-less values. For each tissue, the number of transcript datasets analyzed is indicated in parentheses.

Supplemental Figure 3. Liu *et al.*

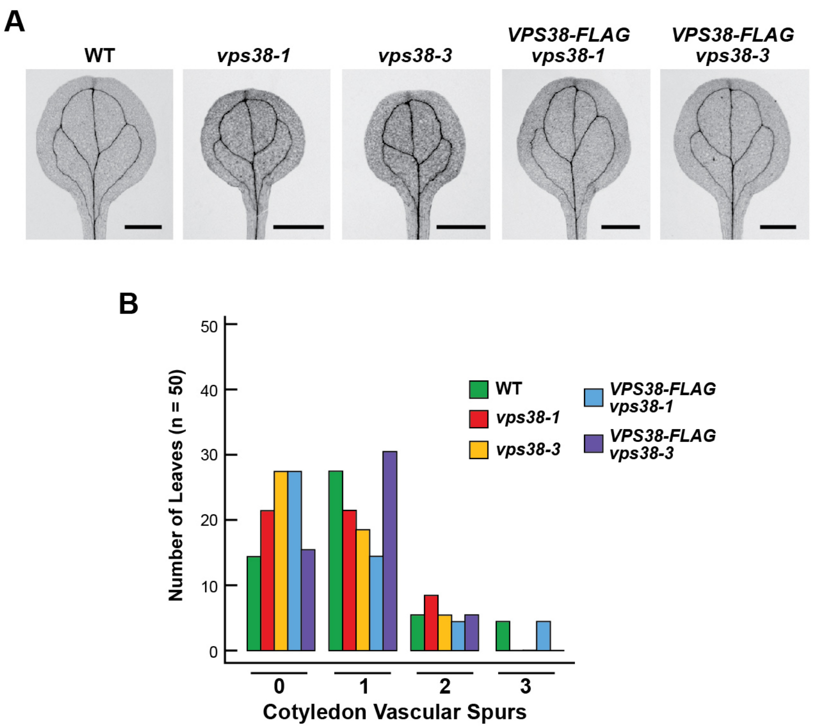

Supplemental Figure 3. Vascular Development is Normal in *vps38* Mutant Cotyledons.

(A) Representative cotyledons from 5-d-old wild-type (WT), *vps38-1*, *vps38-3,* and the *VPS38-FLAG* complementation *Arabidopsis* lines cleared of chlorophyll to show the vascular patterns. Scale bar = 0.5 mm. **(B)** Quantitative distribution of vascular spurs for cotyledons as shown in panel A. Fifty leaves were quantified for each genotype.

Supplemental Figure 4. Liu *et al.*

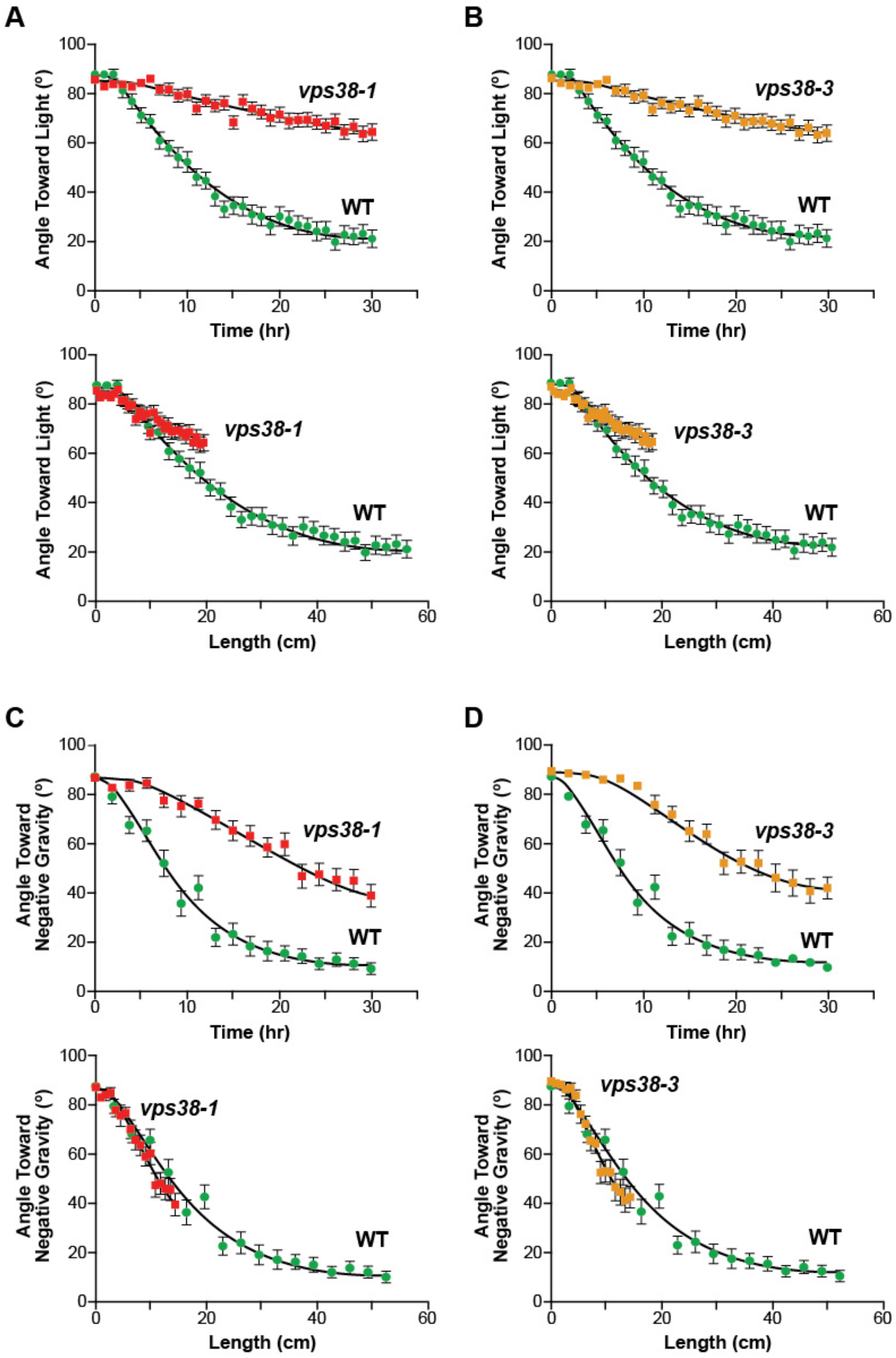

Supplemental Figure 4. VPS38 does not Significantly Impact Hypocotyl Phototropism or Gravitropism. (A,B) Phototropic and **(C,D)** gravitropic responses of hypocotyls. Five-d-old etiolated seedlings of wild type (WT), *vps38-1* **(A,C)**, and *vps38-3* **(B,D)** grown on agar plates were exposed to unilateral white light from the side (100 μmol·m^{-2}·s^{-1}), or rotated 90° by turning the plate. The degrees of hypocotyl curvature (n = 30 seedlings for WT and 20 each for the

vps38 mutants) were measured for each genotype over a 30-hr time course and plotted as the mean (±SE). Upper panels show the actual degrees of bending. Lower panels shows normalization of the responses based on growth rate according to Schöller et al. (2018).

Supplemental Figure 5. Liu *et al.*

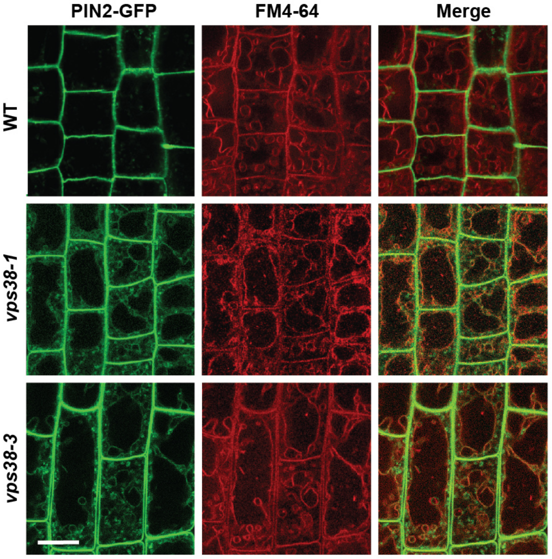

Supplemental Figure 5. Effects of the *vps38* Mutants on the Internalization and Endomembrane Movements of PIN2-GFP and FM4-64. Seven-d-old wild-type, *vps38-1,* and *vps38-3 Arabidopsis* roots expressing PIN2-GFP were incubated in FM4-64 for 6 hr before confocal microscopic imaging. Shown are the fluorescence images from the GFP, FM4-64, and the merge of the two reporters. Scale bar = 20 µm.

Supplemental Figure 6. Liu *et al.*

Original Immunoblot Images of SDS-PAGE gels

Figure 3B

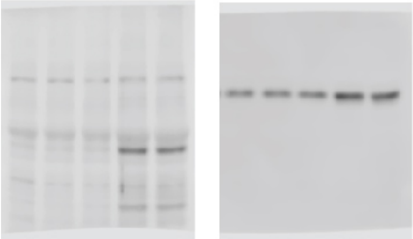

@ FLAG **@PBA1**

Figure 5E

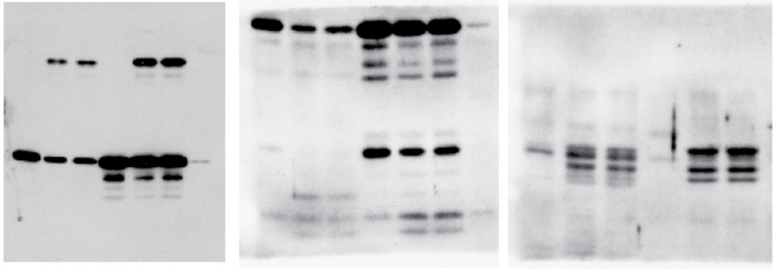

@ 12S **@ 2S** **@VSR1**

Figure 6E

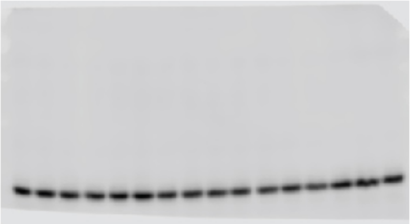

@PBA1

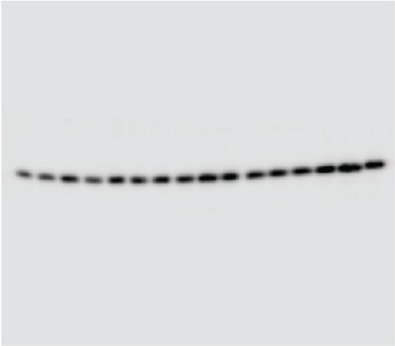

@Histone H3

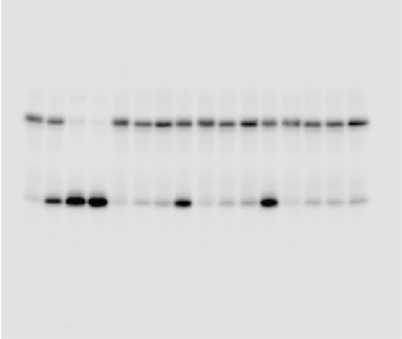

@GFP

Figure 7A

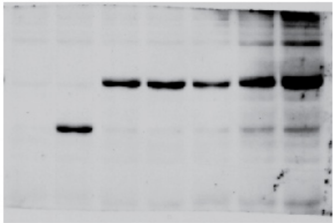

@ATG5

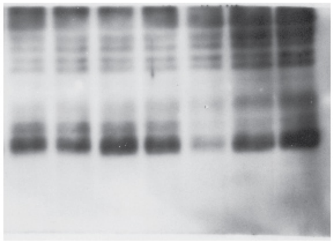

@ATG8

@PBA1

Figure7B

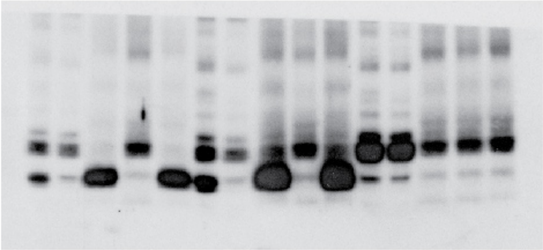

@ATG8
